# A novel kinase regulates dietary restriction-mediated longevity in *Caenorhabditis elegans*

**DOI:** 10.1111/acel.12218

**Published:** 2014-03-21

**Authors:** Manish Chamoli, Anupama Singh, Yasir Malik, Arnab Mukhopadhyay

**Affiliations:** Molecular Aging Laboratory, National Institute of ImmunologyAruna Asaf Ali Marg, New Delhi, 10067, India

**Keywords:** beta-oxidation, *Caenorhabditis elegans*, dietary restriction, fat storage, lifespan, xenobiotic detoxification

## Abstract

Although dietary restriction (DR) is known to extend lifespan across species, from yeast to mammals, the signalling events downstream of food/nutrient perception are not well understood. In *Caenorhabditis elegans*, DR is typically attained either by using the *eat-2* mutants that have reduced pharyngeal pumping leading to lower food intake or by feeding diluted bacterial food to the worms. In this study, we show that knocking down a mammalian MEKK3-like kinase gene, *mekk-3* in *C. elegans*, initiates a process similar to DR without compromising food intake. This DR-like state results in upregulation of beta-oxidation genes through the nuclear hormone receptor NHR-49, a HNF-4 homolog, resulting in depletion of stored fat. This metabolic shift leads to low levels of reactive oxygen species (ROS), potent oxidizing agents that damage macromolecules. Increased beta-oxidation, in turn, induces the phase I and II xenobiotic detoxification genes, through PHA-4/FOXA, NHR-8 and aryl hydrocarbon receptor AHR-1, possibly to purge lipophilic endotoxins generated during fatty acid catabolism. The coupling of a metabolic shift with endotoxin detoxification results in extreme longevity following *mekk-3* knock-down. Thus, MEKK-3 may function as an important nutrient sensor and signalling component within the organism that controls metabolism. Knocking down *mekk-3* may signal an imminent nutrient crisis that results in initiation of a DR-like state, even when food is plentiful.

## Introduction

Dietary restriction (DR) is known to increase lifespan in almost all model systems tested. In mammals, it is associated with health benefits including reduced risk of cancer, neurodegenerative disorders, autoimmune diseases, cardiovascular diseases and type II diabetes (Fontana *et al*., 2010; Omodei & Fontana, 2011; Speakman & Mitchell, 2011). In spite of having positive impact on longevity and quality of life, DR is easier to implement in an experimental model as compared to human beings. DR in humans is challenging in terms of compliance, as we frequently succumb to gastronomic delights which are invariably high in fat and carbohydrates. In this context, it will be beneficial to have a model of DR where, irrespective of the calorie intake, an organism may enjoy its beneficial effects. In this study, we present such a model in *Caenorhabditis elegans*.

Although the beneficial effects of DR are known for decades, the molecular mechanisms and genetic pathways that mediate DR have remained elusive. In this direction, studies using model systems such as *Saccharomyces cerevisiae*, *C. elegans* and *Drosophila* have provided tremendous insights into the mechanisms of DR and longevity (Bishop & Guarente, 2007a; Mair & Dillin, 2008; Fontana *et al*., 2010; Kenyon, 2010). In *C. elegans*, molecular components working downstream of DR are studied after restricting food intake by using either genetic or nongenetic manipulations (Lakowski & Hekimi, 1998; Walker *et al*., 2005; Kaeberlein *et al*., 2006; Bishop & Guarente, 2007b; Panowski *et al*., 2007; Greer & Brunet, 2009). The *eat-2* mutants represent a well-studied genetic model of DR. Due to a mutation in a nonalpha nicotinic acetylcholine receptor subunit, the *eat-2* mutant worms have defective pharyngeal pumping and ingest fewer bacteria (Lakowski & Hekimi, 1998). Several nongenetic methods for DR have also been developed. While some protocols use serial dilutions to essentially limit the amount of bacteria available to the worms, others resort to complete bacterial deprivation (Walker *et al*., 2005; Kaeberlein *et al*., 2006; Bishop & Guarente, 2007b; Panowski *et al*., 2007; Greer & Brunet, 2009). However, these different DR regimes seem to activate distinct pathways. For example, *eat-2* mutants and liquid DR regimes require *pha-4* and *skn-1* transcription factors and are independent of the FOXO transcription factor, *daf-16* (Bishop & Guarente, 2007b; Panowski *et al*., 2007; Park *et al*., 2010). On the other hand, bacterial dilution protocol on solid media requires *daf-16* (Greer & Brunet, 2009). In this context, identification of genes that would induce a DR-like state when manipulated, without the confounding effects of dietary intake, is likely to provide fundamental insights into the mechanisms of DR.

Evolutionary advantages of unpredictable short-term shortage of food may have given rise to the DR response (Masoro, 1996). Molecularly, signalling components within an organism sense low nutrient availability to signal onset of a DR response. In this study, we characterize a novel mammalian MEKK3 (mMEKK3)-like serine–threonine kinase that qualifies as an important component of a nutrient sensing pathway and a DR response initiator. Knocking down *mekk-3* may signal an imminent nutrient crisis that initiates a DR-like state, although food is plentiful and intake is not compromised. This DR-like process dramatically increases lifespan and health span. The increased lifespan is dependent on *pha-4/FOXA* and partially dependent on *skn-1/NRF2*. Importantly, using this model, we further show that *mekk-3* knock-down increases the expression of beta-oxidation genes through the nuclear hormone receptor, NHR-49/HNF4, leading to depletion of stored fat. This metabolic shift towards fatty acid oxidation results in lower reactive oxygen species (ROS) generation without activation of the superoxide dismutases. However, increased fatty acid oxidation, which may lead to the formation of lipophilic endotoxins, activated the xenobiotic detoxification genes such as cytochrome P450 and UDP-glucuronosyltransferase (UGT). We show that this is achieved through the conserved transcription factors, PHA-4/FOXA, NHR-8 and aryl hydrocarbon receptor AHR-1. Thus, our study elucidates a novel mechanism of lifespan extension where a metabolic shift to fatty acid oxidation is coupled to xenobiotic detoxification, leading to dramatic increase in longevity.

## Results

### *mekk-3* dramatically affects lifespan and health span

**Figure 1:**
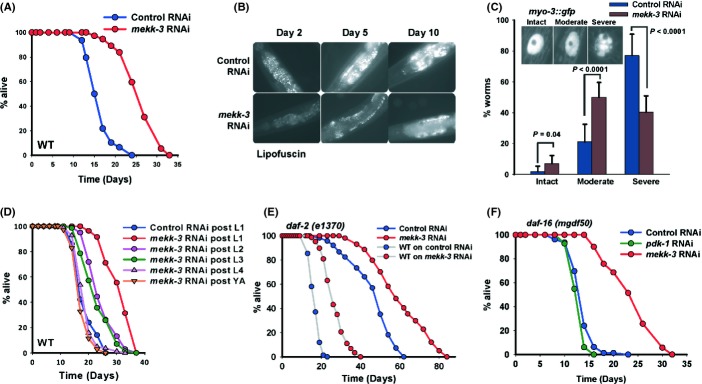
*mekk-3* is an important regulator of lifespan and health span. (A) Lifespan was significantly increased when *mekk-3* was knocked down using RNAi. Mean lifespan (MLS) [days ± SEM, (*n*)] of wild-type (WT) on control RNAi was 16.36 ± 0.34 days (*n* = 78), while on *mekk-3* RNAi it was 26.27 ± 0.52 days (*n* = 74), *P* < 0.0001 by log-rank test. (B) *mekk-3* knock-down delayed accumulation of lipofuscin pigment in the intestine. (C) Muscle nuclei degeneration was delayed in *myo-3::gfp* transgenic worms grown on *mekk-3* RNAi. Nuclei were categorized as intact, moderately or severely degraded according to representative photographs in the inset. Error bars indicate standard deviation; *n* > 20; Student’s *t-*test. (D) Temporal requirements of *mekk-3* knock-down. Maximum extension of lifespan was observed when *mekk-3* RNAi was initiated at L1 (31.40 ± 0.55 days, *n* = 83, *P* < 0.0001), while no extension was seen when it was initiated at L4 (19.16 ± 0.38 days, *n* = 82, *P* = 0.869). As a control, worms were transferred to control RNAi at L1. (E) Knocking down *mekk-3* in *daf-2(e1370)* further increased lifespan. MLS on control RNAi was 46.39 ± 0.85 days (*n* = 165), while on *mekk-3* RNAi was 60.42 ± 1.08 days (*n* = 158), *P* < 0.0001. The grey lines indicate WT on control or *mekk-3* RNAi. (F) *mekk-3* RNAi increased lifespan of *daf-16(mgDf50)*. Mean life-span on control RNAi was 13.86 ± 0.26 days (*n* = 79), whereas on *mekk-3* RNAi it was 24.06 ± 0.59 days (*n* = 70), *P* < 0.0001. *pdk-1* RNAi, which fails to extend life-span in *daf-16(mgDf50)*, was used as control. All life-spans were performed at 20 °C.

In an effort to find kinases that genetically interact with the insulin/IGF-like signalling (IIS) pathway, we performed an RNAi screen to identify *daf-2(e1370)* dauer enhancers (M. Chamoli, A. Mukhopadhyay, unpublished data). We identified *mekk-3* as one of the top candidates (data not shown). Interestingly, *mekk-3* RNAi effectively knocked down the expression of *mekk-3* mRNA (Fig. S1A, Supporting Information) and consistently increased the lifespan of wild-type (WT) N2 Bristol strain by an average of ~60% [mean lifespan (MLS) of WT on control RNAi is 16.36 ± 0.34 days, on *mekk-3* RNAi is 26.27 ± 0.52 days, *P* < 0.0001; Fig. 1A, also see Tables 1 and S1, Supporting Information] without affecting developmental significantly (data not shown). Worms fed RNAi constructs containing either the full-length *mekk-3*cDNA (Fig. S1B) or subcloned fragments (Fig. S1C) also showed increased lifespan. The lifespan extension was not dependent either on addition of FUDR (Fig. S1D), a DNA synthesis inhibitor routinely supplemented during lifespan analysis, or on exposure of worms to OP50 prior to RNAi knock-down using HT115 (Fig. S1E). MEKK-3, encoded by F18F11.5, is a serine–threonine kinase that has 44% homology (26% identity) to mammalian MAP kinase kinase kinase (MAPKKK or MEKK), mMEKK3 and is able to function as a kinase *in vitro* (Fig. S2A,B). The increased longevity in *mekk-3* RNAi worms was distinctly associated with better health. These worms showed delayed as well as lower accumulation of lipofuscin, an age pigment (Fig. 1B). They also maintained healthier musculature, indicated by delayed age-onset nuclear membrane disintegration in muscles (Fig. 1C) and loss of mobility (Fig. S2C), when compared to control RNAi-treated worms. Together, *mekk-3* regulates lifespan and health span in *C. elegans*.

**Table 1:** Summary of lifespan analyses reported in this study

Genetic background	RNAi used	Mean ± SEM (days)	*N*	% change w.r.t control	*P* value
Wild-type	Control	16.36 ± 0.34	78		
	*mekk-3*†	26.27 ± 0.52	74	(+)60.57	< 0.0001
Wild-type	Control	17.37 ± 0.26	111		
	*mekk-3* cDNA construct‡	30.77 ± 0.44	106	(+)77.14	< 0.0001
*daf-2(e1370)*	Control	46.39 ± 0.85	165		
	*mekk-3*	60.42 ± 1.08	158	(+)30.24	< 0.0001
*daf-2(e1368)*	Control	28.34 ± 0.69	119		
	*mekk-3*	40.43 ± 0.84	120	(+)42.66	< 0.0001
*daf-16(mgDf50)*	Control	13.86 ± 0.26	79		
	*mekk-3*	24.06 ± 0.59	70	(+)73.59	< 0.0001
	*pdk-1*	13.07 ± 0.16	82	(−)5.69	≤ 0.001
*daf-3(mgDf90)*	Control	15.93 ± 0.33	90		
	*mekk-3*	25.65 ± 0.48	158	(+)61.01	< 0.0001
*daf-5(e1386)*	Control	14.71 ± 0.36	78		
	*mekk-3*	27.00 ± 0.74	76	(+)83.54	< 0.0001
*daf-2(e1370); daf-3(mgDf90)*	Control	49.86 ± 1.42	78		
	*mekk-3*	73.85 ± 1.18	106	(+)48.11	< 0.0001
*eat-2(ad1116)*	Control	28.94 ± 0.65	90		
	*mekk-3*	27.35 ± 0.61	48	(−)5.49	0.0453
*eat-2(ad1113)*	Control	24.16 ± 0.48	88		
	*mekk-3*	21.66 ± 0.67	62	(−)10.34	0.0089
*eat-2(ad465)*	Control	28.76 ± 0.40	94		
	*mekk-3*	25.64 ± 0.43	100	(−)10.84	< 0.0001
*clk-1(qm30)*	Control	33.04 ± 0.85	67		
	*mekk-3*	34.24 ± 0.83	76	(+)3.63	0.4198
*skn-1(zu169)*	Control	17.86 ± 0.49	76		
	*mekk-3*	26.39 ± 0.79	67	(+)47.76	< 0.0001
*nhr-49(ok2165)*	Control	16.22 ± 0.40	78		
	*mekk-3*	15.49 ± 0.58	45	(−)4.5	0.5572
*nhr-49(nr2041)*	Control	12.91 ± 0.12	141		
	*mekk-3*	13.67 ± 0.29	140	(+)5.88	< 0.0006
*daf-22(m30)*	Control	20.34 ± 0.33	73		
	*mekk-3*	20.59 ± 0.36	51	(+)1.22	0.6539
*mev-1(kn1)*	Control	14.36 ± 0.40	66		
	*mekk-3*	15.06 ± 0.55	52	(+)4.87	0.2868
*gas-1(fc21)*	Control	19.27 ± 0.28	89		
	*mekk-3*	29.74 ± 0.42	66	(+)54.33	< 0.0001
*nhr-8(ok186)*	Control	17.53 ± 0.34	85		
	*mekk-3*	12.71 ± 0.30	42	(−)27.49	≤ 0.001
*aha-1(ok1396)*	Control	16.69 ± 0.33	141		
	*mekk-3*	12.89 ± 0.34	148	(−)22.76	< 0.0001
*ahr-1(ju145)*	Control	16.16 ± 0.45	49		
	*mekk-3*	14.92 ± 0.38	51	(−)7.67	0.0561
*ahr-1(ia3)*	Control	18.43 ± 0.27	134		
	*mekk-3*	20.96 ± 0.41	95	(+)13.72	< 0.0001
*pgp-3(ok3091)*	Control	17.16 ± 0.47	69		
	*mekk-3*	18.63 ± 0.51	72	(+)8.56	0.0915
*pgp-3(pk18)*	Control	17.56 ± 0.30	81		
	*mekk-3*	19.67 ± 0.32	83	(+)12.01	< 0.0001
*fer-15(b26); fem-1(hc17)*	Control	17.82 ± 0.47	115		
	*mekk-3*	24.19 ± 0.63	104	(+)35.74	< 0.0001
Wild-type (15 °C)	Control	19.69 ± 0.46	105		
	*mekk-3*	35.89 ± 1.26	117	(+)82.27	< 0.0001
*hsf-1(sy441)* (15 °C)§	Control	17.85 ± 0.66	73		
	*mekk-3*	31.78 ± 1.69	74	(+)78.03	< 0.0001
Wild-type	Control	18.99 ± 0.44	71		
	*mekk-3*(post-L1)¶	31.40 ± 0.55	83	(+)65.35	< 0.0001
	*mekk-3*(post-L2)	24.87 ± 0.50	99	(+)30.96	< 0.0001
	*mekk-3*(post-L3)	23.40 ± 0.64	70	(+)23.22	< 0.0001
	*mekk-3*(post-L4)	19.16 ± 0.38	82	(+)0.89	0.869
	*mekk-3*(post-YA)	17.81 ± 0.36	70	(−)6.21	0.0336
*smg-1(cc546)*	Control	25.92 ± 0.89	78		
	*mekk-3*	34.60 ± 1.03	77	(+)33.48	< 0.0001
*smg-1(cc546) pha-4(zu225)*††	Control	25.94 ± 0.63	87		
	*mekk-3*	27.84 ± 0.66	79	(+)7.32	0.2621
Wild-type	Control	17.48 ± 0.28	95		
	*nhr-49*	16.80 ± 0.26	125	(−)3.89	0.0944
*eat-2(ad1116)*	Control	24.38 ± 0.41	117		
	*nhr-49*	12.55 ± 0.29	88	(−)48.52	< 0.0001
Wild-type	Control	18.37 ± 0.20	150		
	*nhr-8*	14.44 ± 0.26	106	(−)21.39	< 0.0001
*eat-2(ad1116)*	Control	24.40 ± 0.48	120		
	*nhr-8*	22.52 ± 0.26	124	(−)7.70	< 0.0001
*rde-1(ne219)*	Control	20.01 ± 0.43	106		
	*mekk-3*	20.96 ± 0.45	93	(+)4.74	0.165
*rde-1(ne219); kzIs9*	Control	18.14 ± 0.36	96		
	*mekk-3*	23.37 ± 0.46	93	(+)28.83	< 0.0001
*rde-1(ne219); kzIs20*	Control	16.09 ± 0.51	85		
	*mekk-3*	20.23 ± 0.60	95	(+)25.73	< 0.0001
*rde-1(ne213); kbIs7*	Control	20.16 ± 0.45	86		
	*mekk-3*	19.65 ± 0.50	84	(−)0.02	0.554
Wild-type	Control	18.76 ± 0.35	100		
	*mekk-3* cDNA construct 1	27.98 ± 0.47	117	(+)49.15	< 0.0001
	*mekk-3* cDNA construct 2	27.88 ± 0.39	162	(+)32.71	< 0.0001
	*mekk-3* cDNA construct 3	26.77 ± 0.41	140	(+)42.69	< 0.0001
Wild-type (25 °C)	Control	12.76 ± 0.25	100		
	*mekk-3*	17.68 ± 0.39	108	(+)38.56	< 0.0001
*daf-2(e1370)* (25 °C)‡‡	Control	35.20 ± 0.59	120		
	*mekk-3*	44.41 ± 0.76	120	(+)26.16	< 0.0001
Wild-type (without FUDR)	Control	18.37 ± 0.33	120		
	*mekk-3*	29.19 ± 0.42	120	(+)58.90	< 0.0001
Wild-type (grown on HT115)	Control	18.11 ± 0.33	120		
	*mekk-3*	28.42 ± 0.44	120	(+)56.62	< 0.0001
Wild-type	Control	20.34 ± 0.30	119		
	*mekk-3*	27.01 ± 0.44	107	(+)32.79	< 0.0001
*glp-1(e2141)*§§	Control	24.83 ± 0.42	120		
	*mekk-3*	25.65 ± 0.55	116	(+)3.30	0.0003
Wild-type	Control	18.74 ± 0.43	97		
	*mekk-3*	26.57 ± 0.52	136	(+)41.78	< 0.0001
	Control (5 mm DOG)	23.91 ± 0.63	135		
	*mekk-3* (5 mm DOG)	26.59 ± 0.65	150	(+)11.20	0.0009
Wild-type	Control (OD 3.50)	29.68 ± 1.11	47		
	Control (OD 1.75)	41.47 ± 1.05	49	(+)39.72	< 0.0001
	Control (OD 0.87)	47.92 ± 1.51	51	(+)61.45	< 0.0001
	Control (OD 0.44)	41.73 ± 1.93	49	(+)40.59	< 0.0001
	Control (OD 0.22)	40.89 ± 1.79	46	(+)37.76	< 0.0001
	Control (OD 0.03)	29.27 ± 1.27	49	(−)1.38	0.8725
	*mekk-3* (OD 3.50*)*	34.08 ± 1.73	38		
	*mekk-3* (OD 1.75)	38.07 ± 1.89	43	(+)11.70	0.0296
	*mekk-3* (OD 0.87)	28.40 ± 1.30	45	(−)16.66	0.0027
	*mekk-3* (OD 0.44)	31.10 ± 1.62	41	(−)8.74	0.1619
	*mekk-3* (OD 0.22)	37.14 ± 1.82	37	(+)8.97	0.1251
	*mekk-3* (OD 0.03)	25.86 ± 1.28	44	(−)24.12	< 0.0001

Expanded table for these experiments as well as an independent biological repeat is provided in Table S1.

† All experiments were performed using genomic RNAi construct from Ahringer RNAi library, unless mentioned otherwise.

‡ The full-length cDNA was cloned into pL4440. The cDNA sequence does not match any other sequence in the worm genome.

§ *hsf-1(sy441)* is lethal at higher temperatures. So, the strain and respective control wild-type were grown at 15 °C. Lifespan performed at 15 °C.

¶ *smg-1(cc546); pha-4(zu225)* was grown at 25 °C to deactivate *smg-1*, a component of the NMD pathway. At L4 stage, the strain was shifted to 15 °C for lifespan analysis. At 15 °C, *smg-1* is active and degrades *pha-4* by NMD. The s*mg-1(cc546)* was grown under similar conditions.

†† Eggs were hatched on control RNAi, and worms were then transferred to *mekk-3* RNAi at different larval stages indicated in brackets.

‡‡ The worms were grown at 20 °C till L3 and then transferred to 25 °C for lifespan analysis.

§§ Wild-type and *glp-1(e2141)* worms were hatched at 25 °C and transferred to 20 °C after 24 h. Lifespans were performed at 20 °C.

To increase lifespan, components of the major longevity pathways have to be knocked down at temporally distinct points in the life cycle. For example, reducing insulin/IGF signalling during adulthood or at an advanced age is sufficient to increase lifespan (Dillin *et al*., 2002a). On the other hand, mitochondrial genes have to be knocked down early during the development to positively influence longevity (Dillin *et al*., 2002b). In case of *mekk-3*, knocking the gene down early in life resulted in increased longevity; RNAi initiated at L4 or later had no effect (MLS of WT on control RNAi is 18.99 ± 0.44 days, on *mekk-3* RNAi from L1 is 31.40 ± 0.55 days, *P* < 0.0001, while from L4 is 19.16 ± 0.38 days *P* = 0.869, Fig. 1D, Table 1). Thus, *mekk-3* has temporal requirements that are different from IIS pathway and similar to mitochondrial genes that affect lifespan.

### *mekk-3* works independently of IIS and TGF-beta-like signalling

In *C. elegans*, lifespan and dauer diapause are controlled by two parallel and overlapping signalling cascades, the IIS pathway and the noncanonical TGF-beta-like pathway (Shaw *et al*., 2007; Fielenbach & Antebi, 2008; Kenyon, 2010). Mutation in IIS receptor *daf-2* leads to extended lifespan that is dependent on the FOXO transcription factor DAF-16 and the heat shock transcription factor HSF-1 (Kenyon, 2010). When *daf-2(e1370)* worms were grown on *mekk-3* RNAi at 20 °C, a dramatic increase in lifespan was observed over control RNAi-grown worms, suggesting that MEKK-3 may work in a parallel pathway [MLS of *daf-2(e1370)* on control RNAi is 46.39 ± 0.85 days, on *mekk-3* RNAi is 60.42 ± 1.08 days, *P* < 0.0001; Fig. 1E, Table 1]. RNAi knock-down of *mekk-3* increased lifespan of *daf-2(e1370)* shifted to 25 °C at L3 when IIS completely shuts down, as well as in *daf-2(e1368),* a weaker allele (Fig. S2D,E). It is also possible that *mekk-3* and *daf-2* signalling pathways may overlap. Additionally, the lifespan extension observed on knocking down *mekk-3* requires neither *daf-16* nor *hsf-1* [MLS of *daf-16(mgDf50)* on control RNAi is 13.86 ± 0.26 days, on *mekk-3* RNAi is 24.06 ± 0.59 days, *P* < 0.0001; *hsf-1(sy441)* on control RNAi is 17.85 ± 0.66 days, on *mekk-3* RNAi is 31.78 ± 1.69 days, *P* < 0.0001; Figs 1F and Fig. S3A, Table 1]. *mekk-3* RNAi was also able to extend the lifespans of *daf-3(e1390)* and *daf-5(e1386)* (Fig. S3B,C and Table 1). DAF-3 is a SMAD factor, while DAF-5 is a SNO/SKI transcription factor downstream of the TGF-beta-like pathway (Fielenbach & Antebi, 2008). Further, *mekk-3* RNAi also increased the lifespan of *daf-2(e1370); daf-3(mgDf90)* where the inputs from the DAF-7 pathway into IIS pathway have been eliminated (Padmanabhan *et al*., 2009) (Fig. S3D, Table 1). Taken together, these experiments show that MEKK-3 affects longevity independent of the IIS and TGF-beta-like pathways, possibly using a different mechanism.

### *mekk-3* knock-down induces a DR-like state

**Figure 2:**
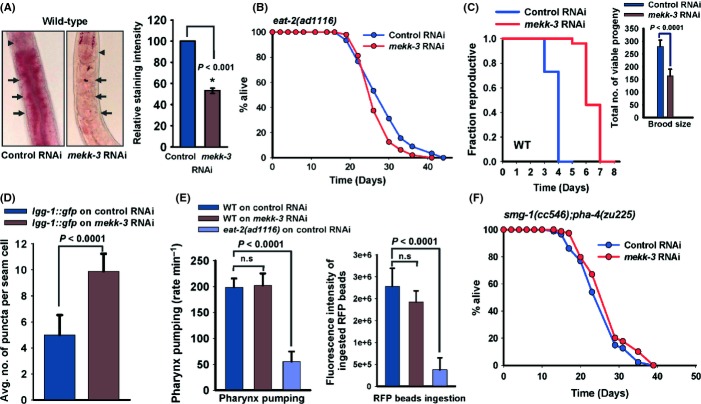
*mekk-3* knock-down initiates a dietary restriction-like state. (A) Oil Red O staining shows that wild-type (WT) worms grown on *mekk-3* RNAi store less fat when compared to control RNAi-grown worms (Left panels). Arrow head indicates the pharynx. Arrows highlight areas showing differences in hypodermal/intestinal fat staining between control and *mekk-3* RNAi. Quantification of staining is presented on the right. Error bars indicate SEM; *n* > 60; Student’s *t-*test). (B) The life-span of *eat-2(ad1116)* was not further increased when *mekk-3* was knocked down. Mean life-span (MLS) on control RNAi was 28.94 ± 0.65 days (*n* = 90), while that on *mekk-3* RNAi was 27.35 ± 0.61 days (*n* = 48), *P* = 0.0453 by log-rank test. Life-spans were performed at 20 °C. (C) Knock-down of *mekk-3* resulted in longer reproductive span [mean reproductive span (MRS) of WT on control RNAi is 2.39 ± 0.14 days, on *mekk-3* RNAi is 3.73 ± 0.19 days; *P* < 0.0001 by log-rank test; *n* = 20] and smaller brood size (inset; brood size 163.86 ± 26.62) on *mekk-3* RNAi compared with control RNAi worms (brood size 279.13 ± 25.33, *P* < 0.0001; Student’s *t-*test; *n* = 15). (D) Enhanced autophagosome formation in hypodermal seam cells in worms grown on *mekk-3* RNAi (9.89 ± 1.35 foci per seam cell) compared with ones grown on control RNAi (4.99 ± 1.53 foci per seam cell) (*P* < 0.001; Student’s *t-*test; *n* > 25, 3–7 seam cells per worm). (E) WT worms grown on *mekk-3* RNAi (pumping rate 210.60 ± 22.35 times per minute) or control RNAi (201.60 ± 14.75 times per minute) have comparable rates of pharyngeal pumping and consequently have similar RFP bead intake (right). *eat-2(ad1116)*, which worms pump slowly (62.40 ± 7.59 times per minute), have lower RFP bead intake (right). (n.s., not significant; *n* > 50 for pharyngeal pumping and *n* > 30 for bead assay; Student’s *t-*test). (F) *mekk-3* RNAi failed to significantly increase lifespan when *pha-4* was mutated. *smg-1(cc546); pha-4(zu225)* worms were grown at 25 °C, and lifespan performed at 15 °C. At 15 °C, *pha-4* is degraded by NMD pathway. MLS of *smg-1(cc546); pha-4(zu225)* grown on control RNAi was 25.94 ± 0.63 days (*n* = 87), on *mekk-3* RNAi was 27.84 ± 0.66 days (*n* = 79), *P* = 0.2621.

As *mekk-3* works independently of the IIS and TGF-beta-like pathway to affect lifespan, we investigated its role in other longevity pathways. In *C. elegans*, the *eat-2* mutants represent a robust genetic model of DR (Lakowski & Hekimi, 1998) that are characterized by long lifespan and reduced fat storage (Lakowski & Hekimi, 1998; Brooks *et al*., 2009). We determined the levels of stored fat in the long-lived *mekk-3* RNAi worms using Oil Red O and Nile Red staining (Yen *et al*., 2010) as well as biochemically by quantifying the triglyceride levels. We found that similar to *eat-2* mutants, *mekk-3* RNAi worms possess considerably fewer fat droplets in the intestinal cells and the hypodermis (Figs 2A and S10A) and had significantly lower triglyceride levels (Fig. S3E). The suppression of fat storage was also substantial when we grew *daf-2(e1370)*, having intrinsically higher fat content, on *mekk-3* RNAi (Figs S3F and S10D). Thus, similar to *eat-2* mutants, *mekk-3* knock-down worms have increased lifespan and low fat reserves.

The *eat-2* mutants (Lakowski & Hekimi, 1998; Brooks *et al*., 2009) as well as *mekk-3* knock-down worms have enhanced longevity and reduced fat storage. So, next we investigated their genetic interactions. We found that *mekk-3* RNAi failed to increase the long lifespans of *eat-2(ad1116)*, *eat-2(ad1113)* and *eat-2(ad465)* to the same extent as in WT [MLS of *eat-2(ad1116)* on control RNAi is 28.94 ± 0.65 days, on *mekk-3* RNAi is 27.35 ± 0.61 days, *P* = 0.0453; Figs 2B and S4A,B, Table 1], suggesting that they may function in the same genetic pathway for lifespan regulation. RNAi of *mekk-3* decreased the low fat storage of *eat-2(ad1116)*, suggesting that metabolic changes precede longevity mechanisms (Figs S4C and S10E). Similar to *eat-2* mutants (Crawford *et al*., 2007; Jia & Levine, 2007), the *mekk-3* RNAi worms have low brood size, longer reproductive span, increased autophagosome formation in the seam cells and smaller body size compared to control RNAi worms (Figs 2C,D and S4D). Additionally, like *eat-2* mutants (Lakowski & Hekimi, 1998), *mekk-3* RNAi did not significantly increase the lifespan of *clk-1(qm30)*, a mutant in the highly conserved demethoxyubiquinone (DMQ) hydroxylase gene that is necessary for the biosynthesis of ubiquinone [MLS of *clk-1(qm30)* on control RNAi is 33.04 ± 0.85 days, while on *mekk-3* RNAi is 34.24 ± 0.83 days, *P* = 0.419, Fig. S4E]. However, the *eat-2* mutants have low pharyngeal pumping rate and as a result ingest less bacteria (Lakowski & Hekimi, 1998). Surprisingly, distinct from *eat-2* mutants, *mekk-3* RNAi did not significantly affect pharyngeal pumping and the worms had normal feeding rate as measured by ingestion of fluorescent beads, GFP-expressing bacteria or by uptake of C_12_ BODIPY (Figs 2E, S12 and S13). Together, *mekk-3* knock-down may initiate a DR-like process by a novel mechanism independent of pharyngeal pumping and feeding defects. It may thus represent a new model of DR.

Dietary restriction may be implemented in *C. elegans* by nongenetic means either by serially diluting the bacteria that the worms feed on (Panowski *et al*., 2007) or by using a nonhydrolysable analogue of glucose (Schulz *et al*., 2007). So, next we studied the genetic interaction of these two DR regimes with the new model of DR. Bacterial dilution-induced DR typically produces a bell-shaped curve when MLSs are plotted against the decreasing values of bacterial concentration (Panowski *et al*., 2007). However, we found that when *mekk-3* RNAi-treated worms were exposed to bacterial dilution starting at adulthood, the MLSs failed to produce a bell-shaped curve (Fig. S5A, Table 1). Additionally, *mekk-3* RNAi failed to extend the lifespan of worms exposed to 2-deoxyglucose (DOG), to the same extent as in untreated worms (Fig. S5B, Table 1). Together, these observations point to the fact that the DR-like state initiated by either knocking down *mekk-3* or through nongenetic means may utilize similar pathways to affect longevity positively.

Genetic and nongenetic models of DR specifically require a FoxA transcription factor, PHA-4 (Panowski *et al*., 2007), to increase lifespan. PHA-4 is not required for IIS pathway-mediated longevity effects. So, next we asked whether our new model of DR requires *pha-4*. We used the temperature-sensitive mutant *smg-1(cc546); pha-4(zu225)* and evaluated whether *mekk-3* RNAi can extend lifespan when *pha-4* is absent; in this mutant, *smg-1* is WT at 15 °C and *pha-4* is degraded by non-sense-mediated decay (NMD) pathway. Under this condition, *mekk-3* failed to increase lifespan significantly [MLS of *smg-1(cc546); pha-4(zu225)* on control RNAi is 25.94 ± 0.63, on *mekk-3* RNAi is 27.84 ± 0.66, *P* = 0.262, Fig. 2F, Table 1]. Thus, similar to *eat-2* mutants and nongenetic liquid DR, knocking down *mekk-3* requires PHA-4 to enhance longevity.

The stress-protective transcription factor SKN-1/NRF2 is important in the ASI neurons to regulate DR-induced longevity, whereas it works downstream of the IIS pathway, in the intestine, to regulate oxidative stress tolerance (Bishop & Guarente, 2007b; Tullet *et al*., 2008; Park *et al*., 2010). We asked whether the new model of DR requires SKN-1. We found that when null mutant *skn-1(zu169)* was grown on *mekk-3* RNAi, lifespan was significantly extended [MLS of *skn-1(zu169)* on control RNAi is 17.86 ± 0.49 days, on *mekk-3* RNAi is 26.39 ± 0.79 days, *P* < 0.0001; Fig. S5C, Table 1], but not to the same extent as WT control (Table 1). Thus, *mekk-3* knock-down-mediated DR partially depends on *skn-1* for extended longevity, much similar to *eat-2* mutant (Park *et al*., 2010) and the nongenetic liquid DR model (Bishop & Guarente, 2007b).

Nutrient signalling is known to play an important role in determining how the reproductive system will influence lifespan (Crawford *et al*., 2007). While DR can extend lifespan in worms lacking the reproductive apparatus, it produced lesser effect in a germline-depleted one (Crawford *et al*., 2007). We asked whether *mekk-3* can extend lifespan in a germline-defective mutant, *glp-1(e2141ts)*. We knocked down *mekk-3* in *glp-1(e2141ts)* and found that the lifespan extension was not to the same extent as in WT [MLS of *glp-1(e2141ts)* on control RNAi 24.83 ± 0.42 days, on *mekk-3* RNAi is 25.65 ± 0.55 days, *P =* 0.003; Fig. S5D]. On the other hand, *mekk-3* RNAi was able to increase lifespan in the sterile strain, *fer-15(b26); fem-1(hc17)* (MLS on control RNAi is 17.82 ± 0.47 days, on *mekk-3* RNAi is 24.19 ± 0.63 days, *P <* 0.0001, Table 1). This suggests that *mekk-3* knock-down genetically interacts with germline in a manner similar to *eat-2(ad1116)*.

Taken together, multiple lines of evidence suggest that knocking down the novel kinase *mekk-3* extends longevity by initiating a DR-like state, similar to the genetic and nongenetic models of DR. However, distinct from the *eat-2* mutants, the induction of this DR-like state is independent of food intake.

### *mekk-3* works in the muscle and hypodermis to regulate lifespan

**Figure 3:**
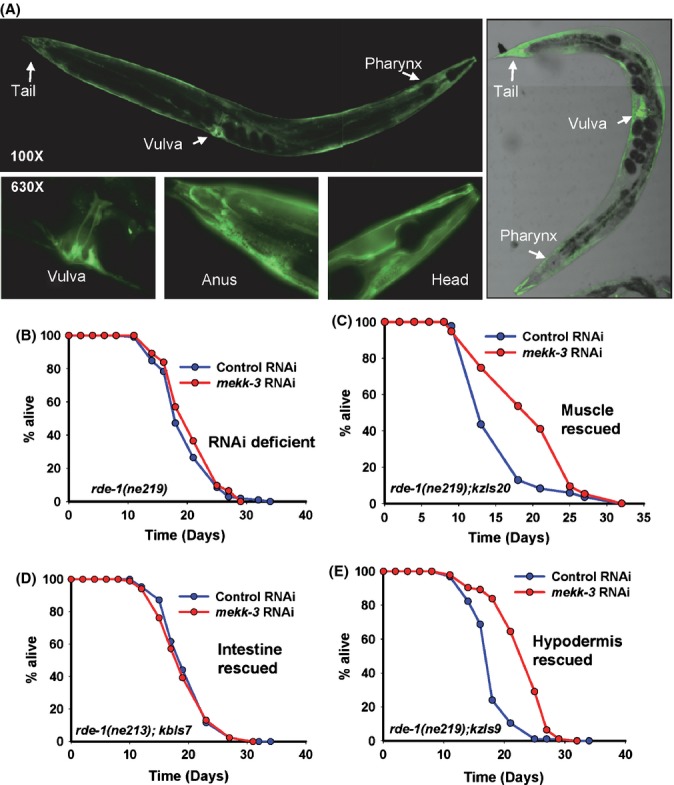
MEKK-3 is required in the hypodermis and muscle for longevity. (A) Expression pattern of *mekk-3p::gfp* transgenic worms. Expression is observed in the hypodermis, vulva and regions adjoining the head and tail. (B) *mekk-3* RNAi had limited effect on the lifespan of RNAi-deficient *rde-1(ne219)*. Mean lifespan (MLS) on control RNAi was 20.01 ± 0.43 (*n* = 106), on *mekk-3* RNAi was 20.96 ± 0.45 days (*n* = 93), *P* = 0.165, log-rank test. (C) *mekk-3* RNAi partially extended the lifespan of *rde-1(ne219); kzIs20*. The *rde-1* gene is rescued in the muscles of *rde-1(ne219)*. Mean lifespan on control RNAi was 16.09 ± 0.51 days (*n* = 85), on *mekk-3* RNAi was 20.23 ± 0.60 days (*n* = 95), *P* < 0.0001. (D) *mekk-3* RNAi had limited effects on lifespan of *rde-1(ne213); kbIs7*. The *rde-1* gene is rescued in the intestine of *rde-1(ne219)*. Mean lifespan on control RNAi was 20.16 ± 0.45 days (*n* = 86), on *mekk-3* RNAi was 19.65 ± 0.50 days (*n* = 84), *P* < 0.554. (E) *mekk-3* RNAi partially extended the lifespan *rde-1(ne219); kzIs9*. The *rde-1* gene is rescued in the hypodermis of *rde-1(ne219)*. Mean lifespan on control RNAi was 18.14 ± 0.36 days (*n* = 96), on *mekk-3* RNAi was 23.37 ± 0.46 days (*n* = 93), *P* < 0.0001. All lifespans were performed at 20 °C.

To study the distribution of *mekk-3* expression, its promoter was cloned in the promoter-less *gfp* vector, pPD95.75. The *mekk-3* promoter drives expression of *gfp* in vulval muscles, body wall muscles, hypodermis, seam cells and tissues adjoining the pharynx and anus (Fig. 3A; Supplementary confocal videos). Expression was also noticed in some neurons but is excluded from the intestine. To find tissues where knocking down of *mekk-3* is sufficient to extend lifespan, we used tissue-specific RNAi systems (Espelt *et al*., 2005; Qadota *et al*., 2007). Interestingly, we found that *mekk-3* RNAi did not extend lifespan when specifically knocked down in the intestine, the major metabolic tissue in the worm. However, partial lifespan extension was observed when expression is reduced in muscles or hypodermis (Fig. 3B–E, Table 1). This suggests that *mekk-3* functions in a cell nonautonomous manner and cooperation between multiple tissues may be required for its normal function. It also establishes hypodermis as an important energy sensing and transducing tissue.

### A metabolic shift towards fatty acid oxidation on *mekk-3* knock-down

*mekk-3* knock-down resulted in increased longevity and decreased fat storage. The decreased fat storage observed may be a result of increased degradation or decreased assimilation of fat, and a gene expression profile may shed light into the mechanism of *mekk-3* knock-down-mediated longevity. We performed a microarray analysis to compare the gene expression profiles of control and *mekk-3* RNAi-treated WT worms. We found that several genes involved in fatty acid degradation and subsequent utilization were upregulated following *mekk-3* knock-down, apart from genes involved in proteolysis and organismal aging (Table 2 and Fig. S11). Genes that were upregulated more than twofolds include lipases, components of the mitochondrial and peroxisomal beta-oxidation system (acyl-CoA synthetase, carnitine palmitoyl transferase, acyl-CoA oxidase, enoyl-CoA hydratase), glyoxylate pathway component (isocitrate/malate synthase) as well as lipid transfer proteins and retinol- /fatty acid-binding proteins. These observations suggested that worms may reprogramme metabolism, following initiation of a DR-like state by *mekk-3* knock-down, towards using beta-oxidation leading to prolonged lifespan. The *eat-2* mutant worms also show similar switch in metabolism (Yuan *et al*., 2012).

**Table 2:** Partial list of genes upregulated on *mekk-3* knock-down as determined by microarray analysis. Genes that may function in lipid metabolism and xenobiotic biotransformation are shown

Gene name	Wormbase ID	Brief descriptions	Fold changes†	*P* value
Lipid metabolism				
*acs-2*	WBGene00009221	Fatty acyl-CoA synthetase	3.7	0.0178
*ech-9*	WBGene00001158	Enoyl-CoA hydratase	170.4	0.0003
*cpt-3*	WBGene00021703	Carnitine palmitoyl transferase	26.2	0.0118
C48B4.1	WBGene00008167	Peroxisomal acyl-CoA oxidase	16.5	0.0005
F25A2.1	WBGene00017764	Lipase	2.6	0.0012
Y49E10.18	WBGene00013037	Lipase	2.6	0.0404
*far-7*	WBGene00001391	Fatty acid- /retinol-binding protein	4.5	0.0277
*gei-7*	WBGene00001564	Isocitrate lyase/malate synthase	2.6	0.0174
C31H5.6	WBGene00007857	Acyl-CoA thioesterase	2.1	0.0020
F47A4.5	WBGene00009801	Involved in lipid catabolic processes	8.5	0.0280
F25E2.3	WBGene00017781	Acyl-CoA thioesterase	2.2	0.0018
*pmp-1*	WBGene00004058	Peroxisomal long-chain fatty acyl transporter	2.0	0.0351
*oac-29*	WBGene00018295	O-Acyltransferase activity	2.8	0.0394
*oac-49*	WBGene00012068	O-Acyltransferase activity	2.6	0.0243
M01A8.1	WBGene00010795	Lipid storage	2.6	0.0240
C30F12.1	WBGene00016260	Lipid storage	2.1	0.0477
*spp-20*	WBGene00005005	Lipid degradation	9.4	0.0010
F47A4.5	WBGene00009801	Lipid transport	8.5	0.028
F09C8.1	WBGene00008621	Phospholipase B1	2.0	0.048
Xenobiotic biotransformation				
*cyp-32B1*	WBGene00021167	Phase I detoxification, cytochrome P450	3.3	0.0134
*cyp-33C8*	WBGene00019967	Phase I detoxification, cytochrome P450	2.6	0.0154
*cyp-34A4*	WBGene00020386	Phase I detoxification, cytochrome P450	3.1	0.0692
*cyp-35A1*	WBGene00015399	Phase I detoxification, cytochrome P450	2.9	0.0075
*cyp-37B1*	WBGene00009226	Phase I detoxification, cytochrome P450	9.7	0.0066
*ugt-16*	WBGene00013901	Phase II detoxification, UDP-glucuronosyltransferase	3.6	0.0186
*ugt-18*	WBGene00013900	Phase II detoxification, UDP-glucuronosyltransferase	820.4	0.0002
*ugt-43*	WBGene00008485	Phase II detoxification, UDP-glucuronosyltransferase	2.1	0.0195

† Fold changes determined between control RNAi and *mekk-3* RNAi-treated worms.

**Figure 4:**
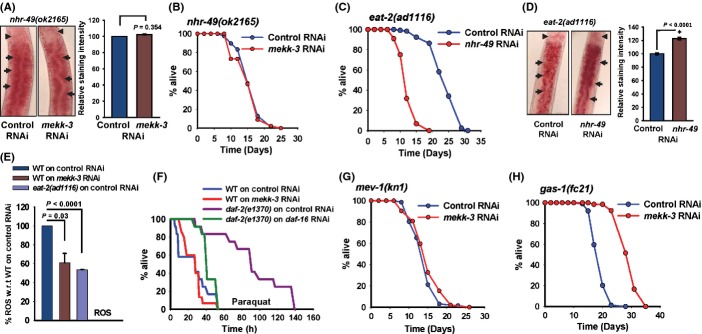
Metabolic reprogramming on *mekk-3* knock-down. (A) *mekk-3* RNAi failed to decrease fat storage in *nhr-49(ok2165)*. Representative worms showing patterns of Oil Red O staining (left) on control and *mekk-3* RNAi. Arrow head indicates the pharynx. Arrows highlight areas showing lack of significant differences in hypodermal/intestinal fat staining between control and *mekk-3* RNAi. Quantification of staining is presented on the right (error bars- SEM; *n* > 50; Student’s *t-*test). (B) *mekk-3* RNAi failed to extend lifespan in *nhr-49(ok2165)*. Mean lifespan (MLS) on control RNAi was 16.22 ± 0.40 days (*n* = 78), while that on *mekk-3* RNAi was 15.49 ± 0.58 days (*n* = 45), *P* = 0.5572 by log-rank test. (C) *nhr-49* RNAi suppressed lifespan of *eat-2(ad1116)*. Mean lifespan on control RNAi was 24.38 ± 0.41 days (*n* = 117), while that on *mekk-3* RNAi was 12.55 ± 0.29 days (*n* = 88), *P* < 0.0001. (D) *eat-2(ad1116)* worms store more fat when grown on *nhr-49* RNAi as compared to control RNAi. Arrows highlight areas showing differences in hypodermal/intestinal fat staining between control and *mekk-3* RNAi. Quantification (right) performed as above; *n* > 50; Student’s *t-*test, *P < 0.0001. (E) Wild-type (WT) worms on *mekk-3* RNAi and *eat-2(ad1116)* on control RNAi have lower reactive oxygen species levels compared with WT grown on control RNAi. Average of three biological replicates; Student’s *t-*test. (F) *mekk-3* RNAi worms were not resistant to external application of 100 mm paraquat. Survival of WT worms on control RNAi was 25.92 ± 4.95 h (*n* = 12), while that on *mekk-3* RNAi was 26.27 ± 2.74 h (*n* = 15) *P* = 0.7432 by log-rank test. The *daf-2(e1370)* worms grown on control RNAi were resistant to paraquat in a *daf-16-*dependent manner. (G) *mekk-3* knock-down failed to extend lifespan of *mev-1(kn1)*. Mean lifespan on control RNAi was 14.36 ± 0.40 days (*n* = 66), while that on *mekk-3*RNAi was 15.06 ± 0.55 days (*n* = 52), *P* = 0.2868. (H) *mekk-3* RNAi extended lifespan of *gas-1(fc21)*. Mean lifespan on control RNAi was 19.27 ± 0.28 days (*n* = 89), while that on *mekk-3*RNAi was 29.74 ± 0.42 days (*n* = 66), *P* < 0.0001. All lifespans were performed at 20 °C.

Nuclear hormone receptor NHR-49, a mammalian HNF4 homolog, transcriptionally regulates many rate-limiting genes of beta-oxidation, including acyl-CoA synthetase, enoyl-CoA hydratase and carnitine palmitoyl transferase (Van Gilst *et al*., 2005; Pathare *et al*., 2012). The lack of beta-oxidation leads to higher amount of stored fat in *nhr-49* mutant worms, as seen in *nhr-49(ok2165)* (Figs S6A and S10F). We reasoned that if *mekk-3* decreases fat storage by upregulating beta-oxidation, it should fail to do so in *nhr-49(ok2165)*. As expected, we found that *mekk-3* RNAi did not affect fat storage in *nhr-49(ok2165)* as determined by Oil Red O, Nile Red and triglyceride quantification (Figs 4A and S10B,G). Consequently, *mekk-3* RNAi failed to extend its lifespan significantly [MLS of *nhr-49(ok2165)* on control RNAi is 16.22 ± 0.40 days, on *mekk-3* 15.49 ± 0.58 days, *P* = 0.56; Fig. 4B, Table 1). The *mekk-3* RNAi was also not able to increase lifespan in another allele of *nhr-49*, namely *nhr-49(nr2041)* to the same extent as in WT (Fig. S6B). In worms, *daf-22* codes for a thiolase required in the last step of peroxisomal beta-oxidation (Zhang *et al*., 2010), an important catabolic step before mitochondrial beta-oxidation of long-chain fatty acids. RNAi of *mekk-3* was unable to increase lifespan in *daf-22(m130)* (Fig. S6C). Taken together, *mekk-3* knock-down-induced DR-like state reprogrammes metabolism towards fatty acid oxidation through NHR-49 and DAF-22 to positively affect lifespan.

Next, we asked whether *eat-2*-mediated DR also requires *nhr-49*-dependent metabolic reprogramming. We grew *eat-2(ad1116)* worms on *nhr-49* RNAi and found that the long lifespan of *eat-2(ad1116)* is significantly suppressed [MLS of *eat-2(ad1116)* on control RNAi is 24.38 ± 0.41 days, on *mekk-3* RNAi is 12.55 ± 0.29 days, *P* < 0.0001; Fig. 4C, Table 1]. The *nhr-49* RNAi had no effect on WT worms (Table 1, also see Table S1). On the other hand, *nhr-49* knock-down increased the fat/triglyceride levels of *eat-2(ad1116)* (Figs 4D and S10C,H). Thus, *nhr-49*-mediated metabolic reprogramming-induced longevity is a common mechanism for lifespan extension during DR.

### Knocking down *mekk-3* generates low ROS and requires Complex II activity

DR is known to produce reduced levels of ROS, potent oxidizing agents that damage cellular macromolecules and may be the underlying cause of aging (Pamplona & Barja, 2007; Page *et al*., 2010). We measured the total cellular ROS in *mekk-3* RNAi worms and found that the levels were significantly lower than that of control RNAi-treated worms, comparable to the ROS generated in *eat-2(ad1116)* (Fig. 4E). It appears that the reduced ROS levels are a result of metabolic shift to beta-oxidation and not due to active ROS detoxification. This is supported by gene expression profiling by microarray and qRT–PCR analysis where none of the five worm superoxide dismutase genes were significantly upregulated when *mekk-3* is knocked down (Fig. S6D). Also, the total cellular SOD activity did not increase when worms were grown on *mekk-3* RNAi (Fig. S6E). Additionally in *nhr-49(ok2165)* worms, that are incapable of making the metabolic shift, *mekk-3* RNAi was not able to significantly decrease the ROS (Fig. S6F). Also, these worms, unlike *daf-2(e1370)*, are not resistant to externally applied paraquat that induces severe oxidative stress (mean survival of WT on control RNAi is 25.92 ± 4.95 h, on *mekk-3* RNAi is 26.27 ± 2.74 h, *P* = 0.7432; *daf-2(e1370)* on control RNAi is 92.58 ± 10.37 h; Fig. 4F). These observations suggest that metabolic reprogramming to fatty acid oxidation intrinsically produces low ROS and contributes to the increased lifespan during *mekk-3* knock-down-mediated DR-like state.

The oxidation of NADH and FADH2 at the mitochondrial ETC. leads to generation of a proton gradient that drives ATP production. The electrons from NADH and FADH2 enter ETC at Complex I and Complex II, respectively. During beta-oxidation, relatively more Complex II is utilized to transfer electrons to ETC, compared to when glucose is the sole source of energy (Mobbs *et al*., 2007). We reasoned that if we disrupt Complex II activity, *mekk-3* knock-down may not be able to extend lifespan. We used a mutation in the *mev-1* gene that codes for cytochrome b in the Complex II. As expected, *mekk-3* RNAi failed to increase the lifespan of *mev-1(kn1)* significantly [MLS of *mev-1(kn1)* on control RNAi is 14.36 ± 0.40 days, on *mekk-3* RNAi is 15.06 ± 0.55 days, *P* = 0.29; Fig. 4G, Table 1]. On the other hand, *mekk-3* RNAi significantly increased the lifespan of *gas-1(fc21),* a mutant in the NADH:ubiquinone oxidoreductase 49 kD subunit of Complex I [MLS of *gas-1(fc21)* on control RNAi is 19.27 ± 0.28 days, on *mekk-3* RNAi is 29.74 ± 0.42 days, *P* < 0.0001; Fig. 4H]. These experiments show that *mekk-3* knock-down that programmes a metabolic shift towards beta-oxidation requires normal Complex II activity to support lifespan extension.

### Knock-down of *mekk-3* activates the xenobiotic detoxification machinery through conserved transcription factors

As observed in dauers, biological systems that depend on fatty acid oxidation for their energy needs activate the xenobiotic detoxification machinery (Wang & Kim, 2003; McElwee *et al*., 2004; Lindblom & Dodd, 2006). Xenobiotic endotoxins are detoxified by a two-step procedure. The phase I detoxification enzymes like cytochrome p450 (CYP) chemically modify offending endotoxins that are then acted upon by phase II enzymes like UGT, making them more soluble (Lindblom & Dodd, 2006). Finally, these modified toxins are secreted out of the cell by ABC transporters (p-glycoproteins) (Sharom, 2011). In the microarray analysis, we found that several genes involved in xenobiotic detoxification, such as CYP and UGT, are significantly upregulated when *mekk-3* is knocked down (Table 2). This indicated that on *mekk-3* knock-down, similar to conditions in dauers, the xenobiotic detoxification machinery may be upregulated to clear internally generated toxins for increased longevity.

**Figure 5:**
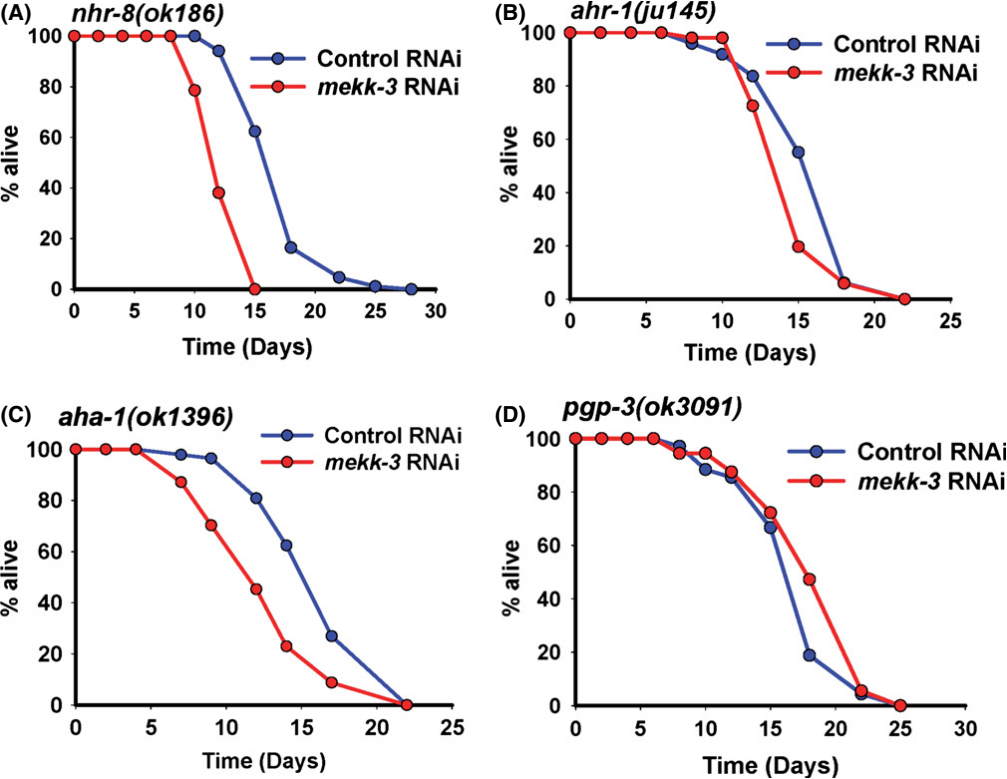
Xenobiotic detoxification is required for *mekk-3* knock-down-mediated longevity. (A) *mekk-3* RNAi did not extend lifespan of *nhr-8(ok186)*. Mean lifespan (MLS) on control RNAi was 17.53 ± 0.34 days (*n* = 85), while that on *mekk-3* RNAi it was 12.71 ± 0.30 days (*n* = 42), *P* = 0.001, by log-rank test. (B) No lifespan extension in *ahr-1(ju145)* on *mekk-3* knock-down. Mean lifespan on control RNAi was 16.16 ± 0.45 days (*n* = 49), while that on *mekk-3* RNAi it was 14.92 ± 0.38 days (*n* = 51), *P* = 0.0561. (C) *mekk-3* RNAi failed to extend lifespan of *aha-1(ok1396)*. Mean lifespan on control RNAi was 16.69 ± 0.33 (*n* = 141), while that on *mekk-3* RNAi it was 12.89 ± 0.34 days (*n* = 148), *P* < 0.0001. (D) No change in lifespan in *pgp-3(ok3091)* worms on *mekk-3* RNAi as compared to control RNAi. Mean lifespan control RNAi was 17.16 ± 0.47 days (*n* = 69), while that on *mekk-3* RNAi it was 18.63 ± 0.51 days (*n* = 72), *P* = 0.0915. All lifespans were performed at 20 °C.

In *C. elegans*, xenobiotic detoxification genes are regulated by NHR-8, a nuclear hormone receptor of the same family as DAF-12 and NHR-48 (Lindblom *et al*., 2001; Lindblom & Dodd, 2006). The expression of some xenobiotic detoxification genes was deregulated in *nhr-8(ok186)* as determined by qRT–PCR (Fig. S7A). So we asked whether *mekk-3* RNAi worms require NHR-8 for their long life. We found that in *nhr-8(ok186)*, knocking down *mekk-3* did not result in increased lifespan [MLS of *nhr-8(ok186)* on control RNAi is 17.53 ± 0.34 days, on *mekk-3* RNAi is 12.71 ± 0.30 days; Fig. 5A, Table 1]. Interestingly, although *nhr-8* plays a crucial role in *mekk-3* knock-down-mediated DR, it appears that *eat-2(ad1116)* does not require *nhr-8* for its increased longevity (Table 1, also see Table S1). The long lifespan on *eat-2(ad1116)* remains unaffected on *nhr-8* RNAi.

In mammals and worms, the CYP genes are regulated by aryl hydrocarbon receptors (AHR) and AHR nuclear translocator (ARNT) (Powell-Coffman *et al*., 1998). *Caenorhabditis elegans* has one gene each for AHR and ARNT, namely *ahr-1* and *aha-1*, respectively; these proteins regulate the expression of some of the xenobiotic genes that were found to be upregulated on *mekk-3* knock-down (Fig. S7B,C). We found that *mekk-3* RNAi failed to extend lifespan in *ahr-1(ju145)*, *ahr-1(ia3)* and *aha-1(ok1396)* to the same extent as in WT worms (Figs 5B,C and S7D, Table 1, see also Table S1). In fact, *mekk-3* RNAi decreased the lifespan of *aha-1(ok1396)* and as observed above, of *nhr-8(ok186)*. We believe that during *mekk-3* knock-down, requirement for xenobiotic detoxification is so enhanced that mutations in key proteins in this pathway may make the worms vulnerable to internally generated toxins and result in suppression of lifespan. It is also possible that these transcription factors have other functions that are required during stresses associated with DR. Further, in ABC transporter (P-glycoproteins) mutants such as *pgp-3(ok3091)* (Fig. 5D) or *pgp-3(pk18)* (Fig. S7E), *mekk-3* RNAi did not extend lifespan to the same extent as in WT.

The lifespan extension by *mekk-3* RNAi requires PHA-4, as shown above. We next asked whether PHA-4 has a role in transcriptional regulation of genes upregulated during this new model of DR. We collated the list of PHA-4 direct targets from modENCODE chromatin immunoprecipitation (ChIP)-sequencing data and compared it with the genes that are upregulated when *mekk-3* is knocked down. We found that over 40% of the genes are common (*P* = 1.83e-9, hypergeometric test), pointing to a direct transcriptional role of PHA-4 in mediating this response (Fig. S8A; Table S2). Additionally, genes involved in xenobiotic detoxification are enriched among the common genes (Fig. S8B). Several representative genes involved in xenobiotic detoxification downstream of *mekk-3* (such as *cyp-32B1*, *37B1* as well as *ugt-16*, *18*) are controlled by PHA-4 as revealed by qRT–PCR analysis (Fig. S8B,C). Interestingly, only a few SKN-1 target genes overlap with *mekk-3* RNAi-upregulated genes and may explain the partial dependence of *skn-1* on *mekk-3* knock-down-mediated lifespan extension (Fig. S8D,E; Table S3). Taken together, these data show that activated xenobiotic detoxification system is an important requirement for *mekk-3* RNAi-induced DR-like state to extend lifespan, and PHA-4, NHR-8 and AHR-1/AHA-1 are important mediators in this process.

### *mekk-3* RNAi-induced metabolic reprogramming upregulates the xenobiotic detoxification genes

**Figure 6:**
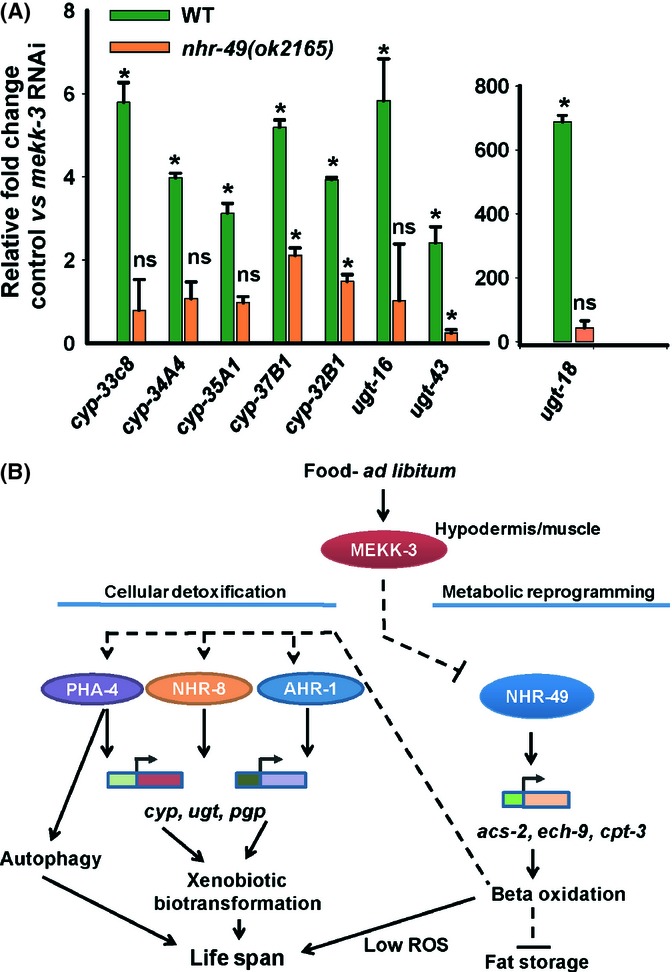
Xenobiotic detoxification genes are triggered by metabolic reprogramming. (A) Transcript levels of select phase I and II xenobiotic detoxification genes were upregulated in wild-type (WT) worms grown on *mekk-3* RNAi (green bar). In *nhr-49(ok2165)* (orange bar), *mekk-3* knock-down failed to increase their levels to the extent of WT. Bars represent transcript abundance in *mekk-3* RNAi worms as compared to control RNAi-grown worms, determined by quantitative RT-PCR. Error bars indicate standard deviation between five biological replicates; *P < 0.05; ns- not significant; Student’s *t-*test between WT and *nhr-49(ok2165)* on control vs. *mekk-3* RNAi for each gene. (B) A mechanistic model showing normal function of MEKK-3. However, knocking down *mekk-3* would induce a metabolic reprogramming where beta-oxidation is upregulated through *nhr-49*. Shifting metabolism to beta-oxidation would produce less reactive oxygen species but would signal the upregulation of xenobiotic detoxification genes through *pha-4*, *nhr-8* and *ahr-1/aha-1*. The xenobiotic biotransformation system would then detoxify lipophilic toxins produced as a result of fatty acid catabolism. PHA-4 may also be responsible for increased autophagy observed in the dietary restriction (DR) worms. Together, the coupling of shift in metabolism and xenobiotic detoxification leads to extended longevity during *mekk-3* knock-down-mediated DR. *acs* = acyl-CoA synthetase, *ech* = enoyl-CoA hydratase, *cpt* = carnitine palmitoyl transferase, *cyp* = cytochrome P450, *ugt* = UDP-glucuronosyltransferase, *pgp* = p-glycoprotein.

Upregulation of the xenobiotic detoxification machinery may either be a direct consequence of *mekk-3* RNAi or an indirect effect of the metabolic shift towards beta-oxidation during DR. To differentiate between these two possibilities, we asked whether *mekk-3* RNAi leads to upregulation of genes involved in xenobiotic response in a *nhr-49* mutant, where beta-oxidation is suppressed. We grew WT and *nhr-49(ok2165)* worms on control or *mekk-3* RNAi and measured the transcript levels of several phase I and II xenobiotic biotransformation genes. As expected, the levels of these genes were significantly increased in WT worms grown on *mekk-3* RNAi (Fig. 6A). Interestingly for all these genes, expression inductions were reduced in *nhr-49(ok2165)* (Fig. 6A), providing evidence that the metabolic reprogramming is responsible for upregulating the xenobiotic biotransformation system in worms where *mekk-3* has been knocked down. Similar results were obtained with *nhr-49(nr2041)* (Fig. S9). Our data suggest that metabolic reprogramming on *mekk-3* knock-down triggers xenobiotic biotransformation gene expression, together leading to long and healthy life.

## Discussion

In this study, we identified a novel mMEKK3-like kinase in *C. elegans* that we speculate may be an important component of a nutrient signalling pathway. Knocking down *mekk-3* leads to phenotypic manifestations and has genetic requirements that are quite similar to DR. Interestingly, the food intake of these worms remain unchanged. However, considering the timing requirements of this gene (Fig. 1D) as well as its epistatic relationship with *clk-1* (Fig. S4E), it is possible that *mekk-3* is a component of the mitochondrial or related longevity pathways. We show that during this DR-like state, two cellular processes are equally important: (i) metabolic shift towards fatty acid oxidation (ii) leading to an increased level of xenobiotic detoxification (Fig. 5B). Together, these two cellular processes create a unique balance in the system that positively affects the lifespan of an organism.

Apart from efficient generation of energy, shifting metabolism from glucose towards lipid oxidation has many other benefits (Mobbs *et al*., 2007; Guarente, 2008). When glucose is used as an energy source, for every FADH2 molecule, five NADH is generated through glycolysis and TCA cycle. On the other hand, during beta-oxidation, the ratio of NADH:FADH2 is only 2:1. The reducing equivalents generated during glucose or lipid breakdown are oxidized in the mitochondrial ETC. Electrons from NADH are transferred to Complex I, while FADH2 is oxidized at Complex II. Complex I is known to produce larger quantities of ROS during the process of transferring electrons to ubiquinone, compared with Complex II (Mobbs *et al*., 2007; Guarente, 2008). As relatively more of Complex II is utilized when lipid is oxidized, compared with when glucose is used, increased beta-oxidation generates lower levels of ROS. This is exactly what we observe on *mekk-3* knock-down and in *eat-2(ad1116)*. As ROS wrecks havoc at the cellular level and have negative impact on normal tissue functioning (Pamplona & Barja, 2007), metabolic reprogramming during DR or in DR-like states is beneficial to an organism and contributes to its increased longevity.

Lipid degradation, however, has a downside; lipophilic toxins may be produced that accumulate in the system (Lindblom & Dodd, 2006). Elevated levels of xenobiotic detoxification enzymes are expressed to counter this issue. In *C. elegans*, such a situation arises in the dauer larvae. During periods of food shortage, worms enter an alternate developmental stage called dauer that have increased fat storage (Fielenbach & Antebi, 2008). Dauer larvae do not feed and depend on lipid catabolism to generate energy. During the dauer stage, elevated expression of xenobiotic detoxification genes are observed (Wang & Kim, 2003; McElwee *et al*., 2004; Lindblom & Dodd, 2006). During the *mekk-3* knock-down-mediated DR-like state, we also found elevated levels of phase I and phase II detoxification genes. Transcription factors controlling these genes, such as PHA-4/FOXA, NHR-8 and AHR-1, were also found to be important during this process. These facts suggest that during DR or in DR-like states, upregulation of xenobiotic detoxification is essential, supporting lifespan extension by lipid oxidation. In fact, the detoxification genes are not upregulated on *mekk-3* knock-down when beta-oxidation is prevented by mutation in *nhr-49*. Recent studies also reflect similar mechanism in mammals. Calorically restricted mice have been shown to upregulate phase I xenobiotic-metabolizing enzymes (Steinbaugh *et al*., 2012), pointing at conservation of this response.

In *C. elegans*, different DR regimes activate alternate pathways and have requirement for different genes. For example, *eat-2* mutants and some nongenetic DR models require *pha-4* and *skn-1* for increased longevity, but do not require *daf-16* (Bishop & Guarente, 2007b; Panowski *et al*., 2007; Park *et al*., 2010). On the other hand, solid dietary restriction (sDR) regimes require *daf-16* (Greer *et al*., 2007). Interestingly, the *mekk-3* knock-down-mediated DR-like state does not require *daf-16*, similar to *eat-2*. However, it requires *pha-4*, but is only partially dependent on *skn-1*. Additionally, although this new model requires NHR-8 for increased lifespan, *eat-2* mutant worms are not affected by *nhr-8* knock-down. It is possible that in *eat-2* mutants, xenobiotic detoxification genes are controlled entirely by SKN-1 (Oliveira *et al*., 2009) or PHA-4, while in our model, this process is under shared regulation. These observations point to the intricate nature of nutrient sensing and differential use of signalling cascades to transduce signals downstream of DR.

Over the last few decades, several theories have evolved to explain causes of aging and lifespan extension. Prominent among them are the mitochondrial free radical theory of aging (MFRTA), mitohormesis and gradual ROS response hypothesis (GRRH) (Pamplona & Barja, 2007; Schulz *et al*., 2007; Hekimi *et al*., 2011). The key difference between MFRTA and mitohormesis/GRRH is in defining the role of ROS. MFRTA proposes that mitochondrial ROS damage macromolecules to cause aging and consequently lowering ROS may increase lifespan. On the other hand, mitohormesis/GRRH requires a burst of ROS production to prime the system to handle subsequent stresses. We found that on *mekk-3* knock-down and in *eat-2(ad1116)*, the worms intrinsically produce lower levels of ROS. At least in the case of *mekk-3* knock-down, this may not be due to higher levels of ROS detoxification machinery within the cells. As determined by microarray and real-time PCR analysis, none of the *sod* genes are significantly upregulated and cellular SOD activity remains unchanged. Although the levels of *ctl-1*, *ctl-2* and *ctl-3* were upregulated several folds (data not shown), this may be attributed to increased peroxisomal beta-oxidation. Consequently, the *mekk-3* knock-down worms do not show hormesis, similar to *eat-2* mutants (Houthoofd *et al*., 2002). Thus, lifespan extension by *mekk-3* knock-down supports MFRTA. Similar observations were made in rodents subjected to DR which showed lower levels of mitoROS, lower resistance to oxidative damage and reduced expression of oxidative defence systems (Pamplona & Barja, 2007), suggesting that such effects of DR may be evolutionarily conserved.

*mekk-3* regulates lifespan in a tissue nonautonomous manner. In line with its expression pattern, *mekk-3* knock-down increases lifespan only when performed in the hypodermis or muscle, but not in the intestine. Thus, MEKK-3 functions in these tissues to regulate metabolism systemically throughout the body, including intestine. Genes expressing in the intestine and neurons have long been known to be major regulators of lifespan and metabolism, especially in case of the IIS pathway (Kenyon, 2010). The cell nonautonomous regulation of longevity by mitochondrial pathway has also been recently described (Durieux *et al*., 2011). Although the intestine is known as the major metabolic tissue (McGhee, 2007), functioning as adipocytes in worms, the hypodermis is now emerging as an important tissue involved in nutrient signalling. The *C. elegans* hypodermis also functions in energy storage and during periods of nutrient deprivation or dauer survival, triglycerides are slowly released from the hypodermal layer that ensure long-term survival (Narbonne & Roy, 2009). This process is controlled by the AMPK signalling cascade (Narbonne & Roy, 2009). Recently, miRNA235 has also been shown to function in the hypodermis to control developmental quiescence depending on nutritional status (Hidefumi *et al*., 2013). Interestingly, the muscle-specific function of *mekk-3* mirrors that of AMPK, a well-established energy sensor (Canto & Auwerx, 2011). Pharmaceutical or genetic activation of AMPK in the muscle leads to increased fatty acid oxidation and glucose uptake (Hardie *et al*., 2006). Thus, it is tempting to speculate that MEKK-3 and AMPK may have overlapping functions in the muscle to regulate metabolism systemically.

MEKK-3 is a negative regulator of lifespan and may constitute a sensory module for nutrient sensing. As it does not express or function in the intestine, it may not be involved directly in the uptake of nutrients. Lowering the levels of MEKK-3 may be speculated to initiate a signalling cascade that signals low nutrient availability, although food intake is normal. This leads to the initiation of a metabolic and physiological reprogramming that benefits the worms in terms of increased lifespan and health span. MEKK-3 is a serine–threonine kinase and has significant homology to mammalian MAPKKK, mMEKK3. It will be interesting to study whether knocking down mMEKK3 can initiate a DR-like state in mammals, without major life style changes.

## Experimental procedures

Detailed materials and methods are reported as Data S1 (Supporting Information). Unless otherwise mentioned, all strains were maintained at 20 °C using standard *C. elegans* techniques, and all RNAi experiments were initiated from eggs. Gravid adult worms were bleached, and the eggs were hatched on plates containing respective RNAi bacteria. L4 or young adult worms were transferred to an intermediate RNAi plate for 12 h and then onto RNAi plates overlaid with 5-fluorodeoxyuridine (FUDR) to a final concentration of 0.1 mg mL^−1^. Worms were scored as dead or alive by tapping them gently with a platinum wire every 2–3 days. Worms that were sick or died from vulval bursting were censored. Statistical analyses for survival were conducted using Mantel–Cox log-rank test using oasis software available at http://sbi.postech.ac.kr/oasis. Lifespan is expressed as average lifespan ± SEM. Full data and additional biological repeat are reported in Table S1. Fat storage was determined in fixed worms using Nile Red or Oil Red O (Yen *et al*., 2010). For estimating pharyngeal pumping, 10–15 young adult worms were videographed for 10 s, and number of pumping was counted in the slowed-down video. To quantify food intake, WT L4 larvae grown on control or *mekk-3* RNAi were placed on NGM plates seeded with a 250:1 (vol:vol) mixture of HT115 bacteria and Fluoresbrites Multifluorescent microspheres (0.2 μm diameter; Polyscience Inc., Warrington, PA, USA). The worms were allowed to feed for 10 min and then washed twice with M9 buffer and photographed. Fluorescent intensities of worms were measured using NIH imagej software. Triglyceride was quantified using a kit (Biovision, Milpitas, CA,USA). Autophagic vesicles were estimated using transgenic worms expressing *lgg-1::gfp*. Microarray analysis was carried out commercially at Genotypic Technology (Bangalore, India) using an Agilent, USA platform. Data were analysed using genespring software (Agilent, Santa Clara, CA, USA) or CLC Genomics Workbench 4 (CLC Bio, Cambridge, MA, USA), and more than twofold expression changes with *P* value < 0.05 is reported. The microarray data are available at GEO repository with series record number GSE40252. Oxidative stress assay was performed in 24-well tissue culture plate containing 1 mL of 100 mm paraquat is reported here. Worms in the paraquat were scored every 2–3 h for survival. Intracellular levels of ROS were determined using 2,7-dichlorofluorescein diacetate (DCF-DA; Molecular Probes, Grand Island, NY, USA).

## Supporting Information

**Fig. S1** (A) *mekk-3* RNAi efficiently knocked down the expression of endogenous *mekk-3* gene, as determined by qRT–PCR. Average of 3 biological replicates; Student’s *t-*test. (B) The *mekk-3* cDNA RNAi construct extended lifespan of WT worms. MLS on control RNAi was 17.37 ± 0.26 days (*n* = 111), on *mekk-3* RNAi was 30.77 ± 0.44 days (*n* = 106), *P* < 0.0001 by Log rank test. (C) The full-length *mekk-3* cDNA was fragmented using restriction endonucleases and subcloned into pL4440. Lifespan analysis using each RNAi clone produced enhanced longevity in WT worms. (D) Knocking down *mekk-3* increased lifespan in WT worms grown in the absence of FUDR. MLS of WT on control RNAi is 18.37 ± 0.33 (*n* = 120) and on *mekk-3* RNAi is 29.14 ± 0.42 (*n* = 120), *P* < 0.0001. (E) The lifespan of WT, grown continuously for two generation on HT115, was also increased when *mekk-3* was knocked down using RNAi. MLS of WT on control RNAi is 18.11 ± 0.33 (*n* = 120) and on *mekk-3* RNAi is 28.42 ± 0.44 (*n* = 120), *P* < 0.0001. Lifespans were performed at 20 °C.

**Fig. S2** (A) Amino acid sequence comparison between *Caenorhabditis elegans* MEKK-3 and mouse mMEKK3. Red arrow indicates Glutamic acid that was mutated to Alanine for the kinase assay. (B) MEKK-3 possesses kinase activity *in vitro* and the activity is dramatically reduced when a glutamic acid at position 123 is mutated to alanine. Kinase assay was carried out using full-length MEKK-3 cDNA construct that was transfected into COS1 cells and immunoprecipitated using anti-Flag antibody. MBP was used as the substrate for the kinases whereas ERK-2 was used as a positive control. Lower panel shows western blot using anti-FLAG antibody. (C) Analysis of age-dependent changes in number of body bends in worms grown on control or *mekk-3*RNAi. Error bars indicate standard deviation. Student’s *t-*test was used to determine statistical significance on each day between control and *mekk-3* RNAi-treated worms, (*n* > 40). (D) Knocking down *mekk-3* in *daf-2(e1370)* grown at 25 °C further increased lifespan. These worms were grown at 20 °C till L3 and shifted to 25 °C for lifespan analysis. MLS of *daf-2(e1370)* on control RNAi is 35.20 ± 0.59 (*n* = 120) and on *mekk-3* RNAi is 44.41 ± 0.76 (*n* = 120), *P* < 0.0001 by Log rank test. (E) Knocking down *mekk-3* further increased the lifespan in *daf-2(e1368)* grown at 20 °C. MLS of *daf-2(e1368)* on control RNAi is 28.34 ± 0.69 (*n* = 119) and on *mekk-3* RNAi is 40.43 ± 0.84 (*n* = 120), *P* < 0.0001.

**Fig. S3** (A) *mekk-3* RNAi extended lifespan of *hsf-1(sy441)*. MLS on control RNAi was 17.85 ± 0.66 days (*n* = 73), on *mekk-3* RNAi was 31.78 ± 1.69 days (*n* = 74), *P* < 0.0001, Log rank test. Lifespans were performed at 15 °C. (B) *mekk-3* RNAi increased lifespan of *daf-3(mgDf90)*. MLS on control RNAi was 15.93 ± 0.33 (*n* = 90), on *mekk-3* RNAi was 25.65 ± 0.48 days (*n* = 158), *P* < 0.0001. Lifespans were performed at 20 °C. (C) *mekk-3* RNAi extended lifespan of *daf-5(e1386)*. MLS on control RNAi was 14.71 ± 0.36 days (*n* = 78), on *mekk-3* RNAi was 27.00 ± 0.74 days (*n* = 76), *P* < 0.0001. Lifespans were performed at 20 °C. (D) *mekk-3* RNAi increased lifespan of *daf-2(e1370); daf-3(mgDf90)*. MLS of on control RNAi was 49.86 ± 1.42 days (*n* = 78), on *mekk-3* RNAi was 73.85 ± 1.18 days (*n* = 106), *P* < 0.0001. Lifespans were performed at 20 °C. (E) Levels of triglyceride were measured biochemically in worms grown on control RNAi or *mekk-3* RNAi. The *eat-2(ad1116)* worms that are known to store less fat was taken as a control for the experiment. Average of three independent biological replicates; error bars-standard deviation; Student’s *t-*test. (F) Decreased fat storage of *daf-2(e1370)* when grown on *mekk-3* RNAi compared to control RNAi-grown worms as determined by Oil Red O staining (left). Arrow head indicates the pharynx. Arrows highlight areas showing differences in hypodermal/intestinal fat staining between control and *mekk-3* RNAi. Quantification of staining intensity is presented on right. Error bars indicate SEM; *n* > 50; Student’s *t-*test. Worms were maintained at 20 °C.

**Fig. S4** (A) Lifespan of *eat-2(ad1113)* on *mekk-3* RNAi. MLS on control RNAi was 24.16 ± 0.48 days (*n* = 88), on *mekk-3* RNAi was 21.66 ± 0.67 days (*n* = 62), *P* = 0.0089 by Log rank test. (B) Lifespan of *eat-2(ad465)* on *mekk-3* RNAi. MLS on control RNAi was 28.76 ± 0.76 days (*n* = 94), on *mekk-3*RNAi was 25.64 ± 0.43 days (*n* = 100), *P* < 0.0001. (C) Oil Red O staining of fat stored in *eat-2(ad1116)* grown on control or *mekk-3* RNAi. Quantitation on right; *n* > 50; Student’s *t-*test. (D) WT worms grown on *mekk-3* RNAi have smaller size compared to control RNAi-grown worms. Error bars indicate SEM; *n* > 40; Student’s *t-*test. (E) *mekk-3* RNAi failed to extend lifespan of *clk-1(qm30)*. MLS on control RNAi was 33.04 ± 0.85 days (*n* = 67), on *mekk-3* RNAi was 34.24 ± 0.83 days (*n* = 76), *P* = 0.4198. All lifespan were performed at 20 °C.

**Fig. S5** (A) WT worms grown on control or *mekk-3* RNAi were transferred to liquid DR culture (see Data S1) having different concentrations of HT115 bacteria. The typical bell-shaped curve obtained after exposing WT worms, that were grown on control RNAi, to DR is absent in *mekk-3* RNAi-treated worms. Mean lifespan is plotted against decreasing concentration of bacteria; error bars represent SEM. (B) The *mekk-3* RNAi is unable to increase the lifespan of WT worms treated with 2-deoxy glucose (DOG) to the extent observed in untreated worms. In presence of 5 mm 2-deoxy glucose, MLS of WT on control RNAi was 23.91 ± 0.63 days (*n* = 135), on *mekk-3* RNAi was 26.59 ± 0.65 days (*n* = 150) *P* < 0.0009. The grey lines indicate untreated WT worms grown on control or *mekk-3* RNAi. (C) Lifespan of *skn-1(zu169)* on *mekk-3* RNAi. MLS on control RNAi was 17.86 ± 0.49 days (*n* = 76), on *mekk-3* RNAi was 26.39 ± 0.79 days (*n* = 67) *P* < 0.0001. The grey lines indicate WT grown on control or *mekk-3* RNAi. Lifespans were performed at 20 °C. (D) The *mekk-3* RNAi did not further increase the lifespan of *glp-1(e2141)*. MLS on control RNAi was 24.83 ± 0.42 days (*n* = 120), on *mekk-3* RNAi was 25.65 ± 0.55 days (*n* = 116) *P* = 0.0003. The grey lines indicate WT grown on control or *mekk-3* RNAi. WT and *glp-1(e2141)* worms were hatched at 25 °C and transferred to 20 °C after 24 h. Lifespans were performed at 20 °C.

**Fig. S6** (A) *nhr-49(ok2165)* worms have higher amount of stored fat as indicated by Oil red O staining. Arrow head indicates the pharynx. Arrows highlight areas showing differences in hypodermal/intestinal fat staining between control and *mekk-3* RNAi. Quantitation of staining is shown in the right panel. Error bar- SEM; *n* > 50; Student’s *t*-test. (B) *mekk-3* RNAi was not able to increase lifespan in *nhr-49(nr2041)* to the same extent as in WT. MLS on control RNAi was 12.91 ± 0.12 (141), on *mekk-3* RNAi was 13.67 ± 0.29 (140), *P* < 0.0006. Lifespans were performed at 20 °C. (C) *mekk-3*RNAi failed to increase lifespan in *daf-22(m30)*. MLS on control RNAi was 20.34 ± 0.33 (*n* = 73), on *mekk-3* RNAi was 20.59 ± 0.36 (*n* = 51), *P* = 0.6539. Lifespans were performed at 20 °C. (D) The expression of *sod* genes did not increase significantly between control- and *mekk-3* RNAi-treated WT worms. The data for each of the *sod* genes is normalized to its expression in control RNAi-treated worms. Error bars indicate standard deviation over five independent biological replicates. *P* values- Student’s *t-*test between control and *mekk-3* RNAi-treated worms for each gene. (E) The total SOD activity did not increase when WT worms were grown on *mekk-3* RNAi. A *daf-2(e1370)* mutant had high levels of SOD activity. Graph represents average of 3 biological replicates; **P* < 0.01, Student’s *t-*test between indicated samples. (F) *mekk-3* RNAi decreased ROS in WT but failed to decrease it in *nhr-49(ok2165)*. Average of three biological replicates. Error bars indicate standard deviation. Student’s *t-*test between samples indicated by lines.

**Fig. S7** The expression of several xenobiotic detoxification genes are deregulated in (A) *nhr-8(ok186)*, (B) *aha-1(ok1396)* and (C) *ahr-1(ju145)* mutants. Error bars- Standard deviation; *P* < 0.05, ns- not significant; Student’s *t-*test between WT and respective mutants for each gene. (D) Lifespan of *ahr-1(ia3)* on *mekk-3* RNAi. MLS on control RNAi was 18.43 ± 0.27 days (*n* = 134), on *mekk-3* RNAi was 20.96 ± 0.41 days (*n* = 95), *P* < 0.0001 by Log rank test. (E) Lifespan of *pgp-3(pk18)* on *mekk-3* RNAi. MLS on control RNAi was 17.56 ± 0.30 days (*n* = 81), on *mekk-3* RNAi was 19.67 ± 0.32 days (*n* = 83), *P* < 0.0001. All lifespans were performed at 20 °C.

**Fig. S8** (A) Venn diagram showing the overlap of PHA-4 direct target genes (ModENCODE) that are upregulated when WT worms are grown on *mekk-3* RNAi (overlap 40.8%, *P* = 1.83e-9, hypergeometric test). See Table S2 for gene-list. (B) Categorization of the PHA-4 target genes that are upregulated on *mekk-3* RNAi using DAVID (*david.abcc.ncifcrf.gov*). (C) Several xenobiotic detoxification genes that are upregulated on *mekk-3* RNAi in WT worms are downregulated when *pha-4* is also knocked down. Worms were either grown on Control RNAi, *mekk-3* RNAi or initiated on *mekk-3* RNAi and transferred to *pha-4* RNAi post-L4. Error bars- Standard deviation; *P* < 0.05, ns- not significant; Student’s *t-*test between WT grown on control RNAi vs. ones grown on *mekk-3* or *mekk-3 + pha-4* RNAi for each gene. (D) Venn diagram showing the overlap of direct target genes of SKN-1 (ModENCODE) that are upregulated when WT worms are grown on *mekk-3* RNAi (overlap 7.5%, *P* = 0.0086, hypergeometric test). See Table S3A for gene-list. (E) Venn diagram showing the overlap of SKN-1 target genes (from microrarray, Oliveira *et al*., 2009) that are upregulated when WT worms are grown on *mekk-3* RNAi (overlap 4.3%, *P* = 0.00009, hypergeometric test). See Table S3B for gene-list.

**Fig. S9** Transcript levels of select phase I and II xenobiotic detoxification genes were upregulated in WT worms undergoing *mekk-3* knock-down-mediated DR (Green bar).

**Fig. S10** Nile Red staining of fixed WT or mutant worms grown on Control or specific RNAi. Left panel shows representative photo of worms while right panel shows quantification of fluorescence.

**Fig. S11** The genes upregulated when WT worms were grown on *mekk-3* RNAi were categorized on the basis of GO term using DAVID and REVIGO softwares; details in Data S1.

**Fig. S12** Wild-type worms on Control or *mekk-3*RNAi as well as *eat-2(ad1116)* grown on control RNAi were fed BODIPY containing bacterial feed for 20 min.

**Fig. S13** The *mekk-3* RNAi worms have normal bacterial food intake.

**Data S1** Experimental procedures.

**Table S1** Complete lifespan data.

**Table S2** List of PHA-4 target genes that are upregulated when WT is grown on mekk-3 RNAi.

**Table S3** (A) SKN-1 direct target genes (obtained from ModENCODE) that are upregulated in WT grown on mekk-3 RNAi. (B) SKN-1 target genes (determined by microarray; see reference) that are upregulated in WT grown on mekk-3 RNAi.

**Video S1** Confocal images of the head region of a *mekk-3::gfp* transgenic worm, stacked in Z plane to create a movie.

**Video S2** Confocal images of the tail region of a *mekk-3::gfp* transgenic worm, stacked in Z plane to create a movie.

**Video S3** Confocal images of the vulval region of a *mekk-3::gfp* transgenic worm, stacked in Z plane to create a movie.
