# Supplementary figures and images for: A novel kinase regulates dietary restriction-mediated longevity in Caenorhabditis elegans
Source: Aging Cell. 2014 Mar 21;13(4):641–55. doi: 10.1111/acel.12218 (PMC4326946; doi:10.1111/acel.12218)

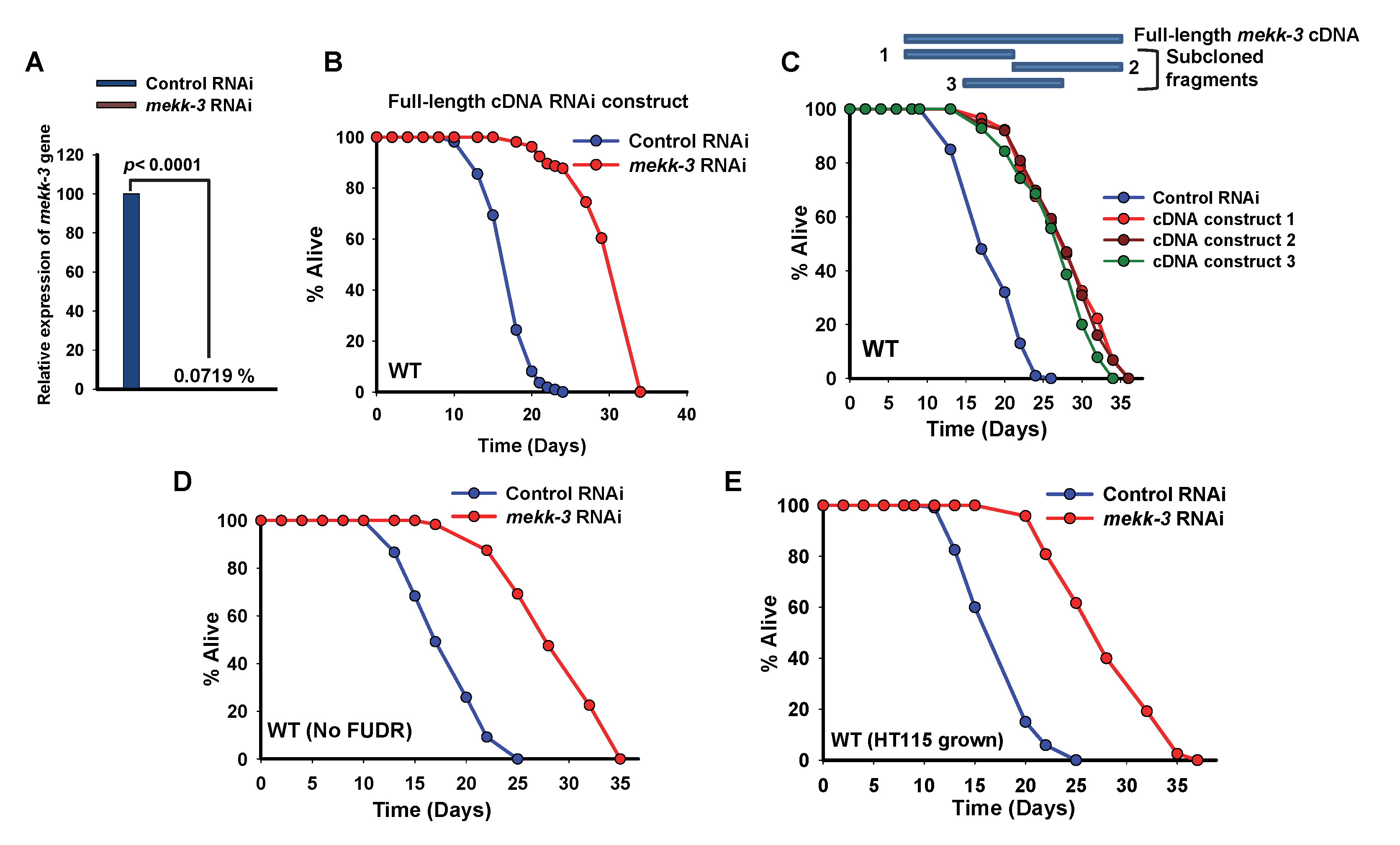

Supplement: Supplementary file 1 — Fig. S1 (A) mekk-3 RNAi efficiently knocked down the expression of endogenous mekk-3 gene, as determined by qRT–PCR. Average of 3 biological replicates; Student’s t-test. (B) The mekk-3 cDNA RNAi construct extended lifespan of WT worms. MLS on control RNAi was 17.37 ± 0.26 days (n = 111), on mekk-3 RNAi was 30.77 ± 0.44 days (n = 106), P < 0.0001 by Log rank test. (C) The full-length mekk-3 cDNA was fragmented using restriction endonucleases and subcloned into pL4440. Lifespan analysis using each RNAi clone produced enhanced longevity in WT worms. (D) Knocking down mekk-3 increased lifespan in WT worms grown in the absence of FUDR. MLS of WT on control RNAi is 18.37 ± 0.33 (n = 120) and on mekk-3 RNAi is 29.14 ± 0.42 (n = 120), P < 0.0001. (E) The lifespan of WT, grown continuously for two generation on HT115, was also increased when mekk-3 was knocked down using RNAi. MLS of WT on control RNAi is 18.11 ± 0.33 (n = 120) and on mekk-3 RNAi is 28.42 ± 0.44 (n = 120), P < 0.0001. Lifespans were performed at 20 °C. [file acel0013-0641-sd1.tiff]

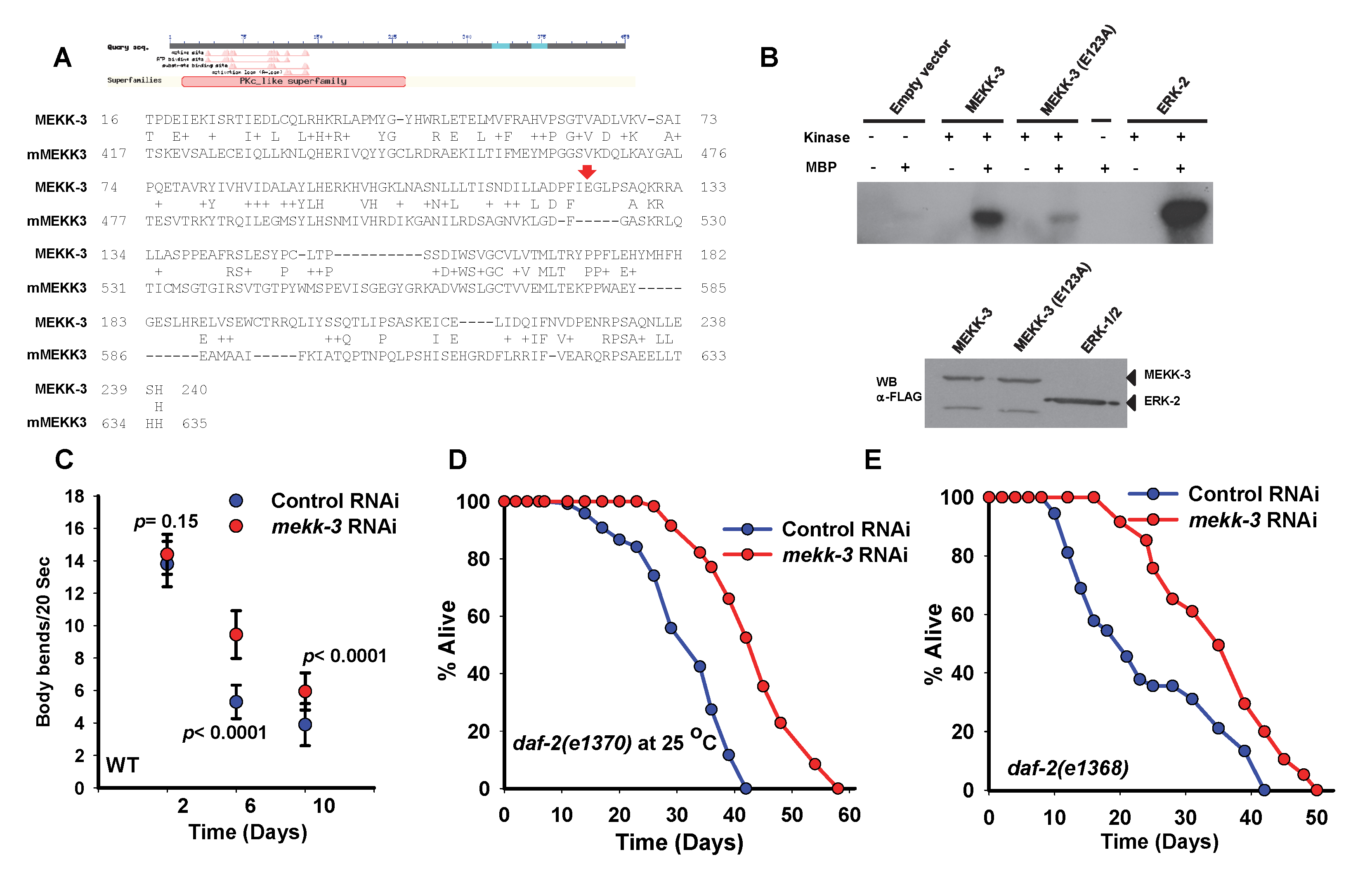

Supplement: Supplementary file 2 — Fig. S2 (A) Amino acid sequence comparison between Caenorhabditis elegans MEKK-3 and mouse mMEKK3. Red arrow indicates Glutamic acid that was mutated to Alanine for the kinase assay. (B) MEKK-3 possesses kinase activity in vitro and the activity is dramatically reduced when a glutamic acid at position 123 is mutated to alanine. Kinase assay was carried out using full-length MEKK-3 cDNA construct that was transfected into COS1 cells and immunoprecipitated using anti-Flag antibody. MBP was used as the substrate for the kinases whereas ERK-2 was used as a positive control. Lower panel shows western blot using anti-FLAG antibody. (C) Analysis of age-dependent changes in number of body bends in worms grown on control or mekk-3RNAi. Error bars indicate standard deviation. Student’s t-test was used to determine statistical significance on each day between control and mekk-3 RNAi-treated worms, (n > 40). (D) Knocking down mekk-3 in daf-2(e1370) grown at 25 °C further increased lifespan. These worms were grown at 20 °C till L3 and shifted to 25 °C for lifespan analysis. MLS of daf-2(e1370) on control RNAi is 35.20 ± 0.59 (n = 120) and on mekk-3 RNAi is 44.41 ± 0.76 (n = 120), P < 0.0001 by Log rank test. (E) Knocking down mekk-3 further increased the lifespan in daf-2(e1368) grown at 20 °C. MLS of daf-2(e1368) on control RNAi is 28.34 ± 0.69 (n = 119) and on mekk-3 RNAi is 40.43 ± 0.84 (n = 120), P < 0.0001. [file acel0013-0641-sd2.tiff]

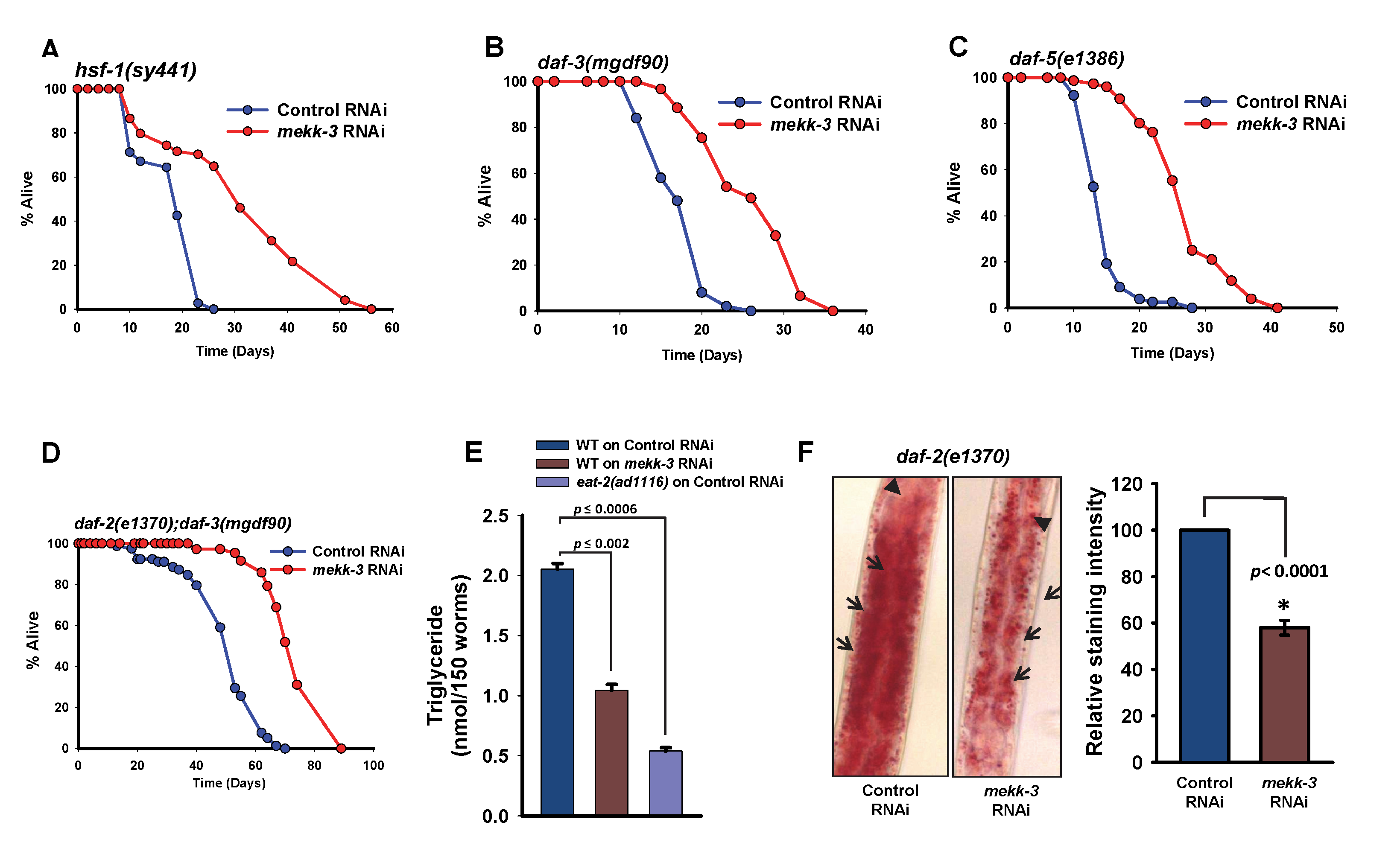

Supplement: Supplementary file 3 — Fig. S3 (A) mekk-3 RNAi extended lifespan of hsf-1(sy441). MLS on control RNAi was 17.85 ± 0.66 days (n = 73), on mekk-3 RNAi was 31.78 ± 1.69 days (n = 74), P < 0.0001, Log rank test. Lifespans were performed at 15 °C. (B) mekk-3 RNAi increased lifespan of daf-3(mgDf90). MLS on control RNAi was 15.93 ± 0.33 (n = 90), on mekk-3 RNAi was 25.65 ± 0.48 days (n = 158), P < 0.0001. Lifespans were performed at 20 °C. (C) mekk-3 RNAi extended lifespan of daf-5(e1386). MLS on control RNAi was 14.71 ± 0.36 days (n = 78), on mekk-3 RNAi was 27.00 ± 0.74 days (n = 76), P < 0.0001. Lifespans were performed at 20 °C. (D) mekk-3 RNAi increased lifespan of daf-2(e1370); daf-3(mgDf90). MLS of on control RNAi was 49.86 ± 1.42 days (n = 78), on mekk-3 RNAi was 73.85 ± 1.18 days (n = 106), P < 0.0001. Lifespans were performed at 20 °C. (E) Levels of triglyceride were measured biochemically in worms grown on control RNAi or mekk-3 RNAi. The eat-2(ad1116) worms that are known to store less fat was taken as a control for the experiment. Average of three independent biological replicates; error bars-standard deviation; Student’s t-test. (F) Decreased fat storage of daf-2(e1370) when grown on mekk-3 RNAi compared to control RNAi-grown worms as determined by Oil Red O staining (left). Arrow head indicates the pharynx. Arrows highlight areas showing differences in hypodermal/intestinal fat staining between control and mekk-3 RNAi. Quantification of staining intensity is presented on right. Error bars indicate SEM; n > 50; Student’s t-test. Worms were maintained at 20 °C. [file acel0013-0641-sd3.tiff]

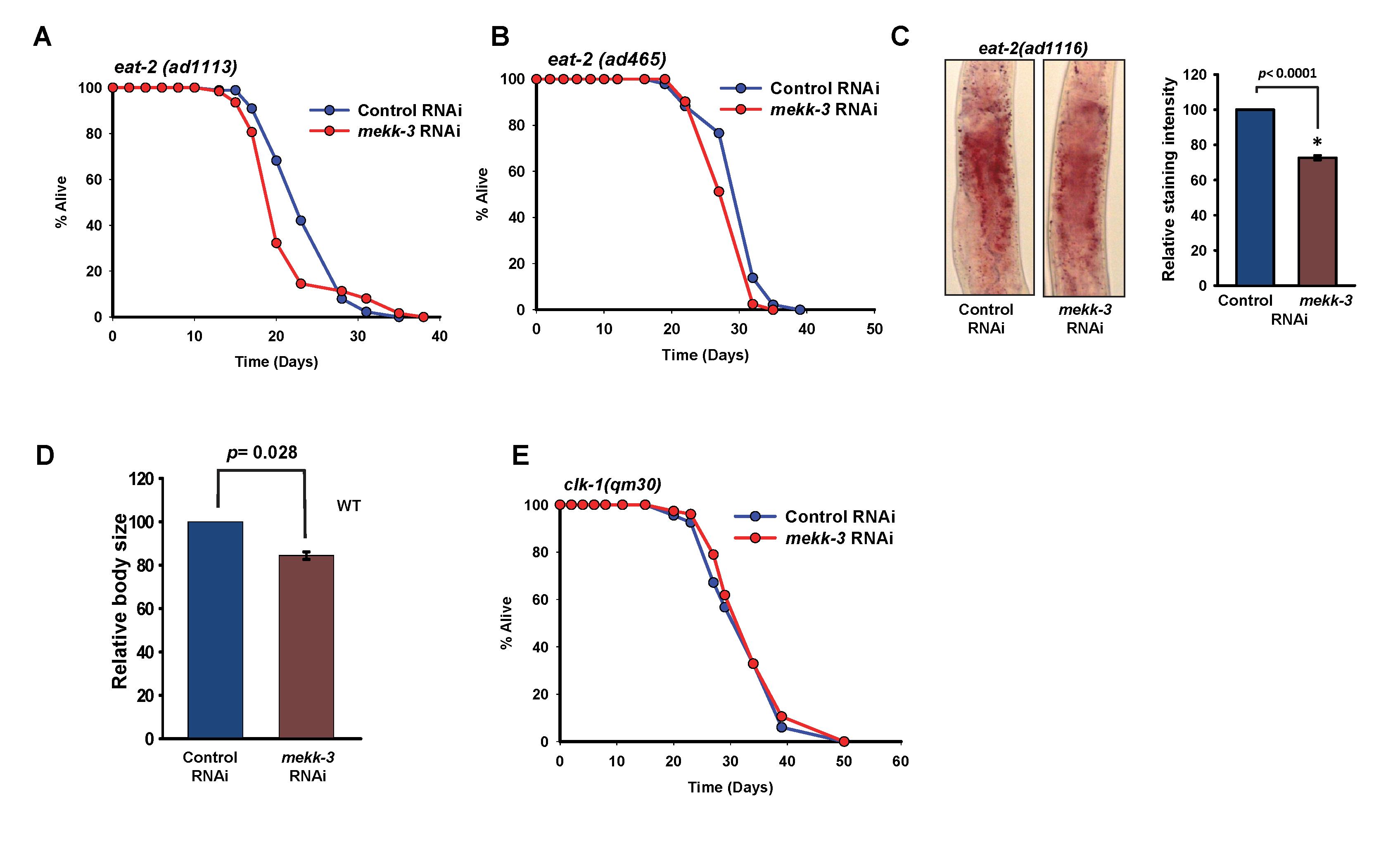

Supplement: Supplementary file 4 — Fig. S4 (A) Lifespan of eat-2(ad1113) on mekk-3 RNAi. MLS on control RNAi was 24.16 ± 0.48 days (n = 88), on mekk-3 RNAi was 21.66 ± 0.67 days (n = 62), P = 0.0089 by Log rank test. (B) Lifespan of eat-2(ad465) on mekk-3 RNAi. MLS on control RNAi was 28.76 ± 0.76 days (n = 94), on mekk-3RNAi was 25.64 ± 0.43 days (n = 100), P < 0.0001. (C) Oil Red O staining of fat stored in eat-2(ad1116) grown on control or mekk-3 RNAi. Quantitation on right; n > 50; Student’s t-test. (D) WT worms grown on mekk-3 RNAi have smaller size compared to control RNAi-grown worms. Error bars indicate SEM; n > 40; Student’s t-test. (E) mekk-3 RNAi failed to extend lifespan of clk-1(qm30). MLS on control RNAi was 33.04 ± 0.85 days (n = 67), on mekk-3 RNAi was 34.24 ± 0.83 days (n = 76), P = 0.4198. All lifespan were performed at 20 °C. [file acel0013-0641-sd4.tiff]

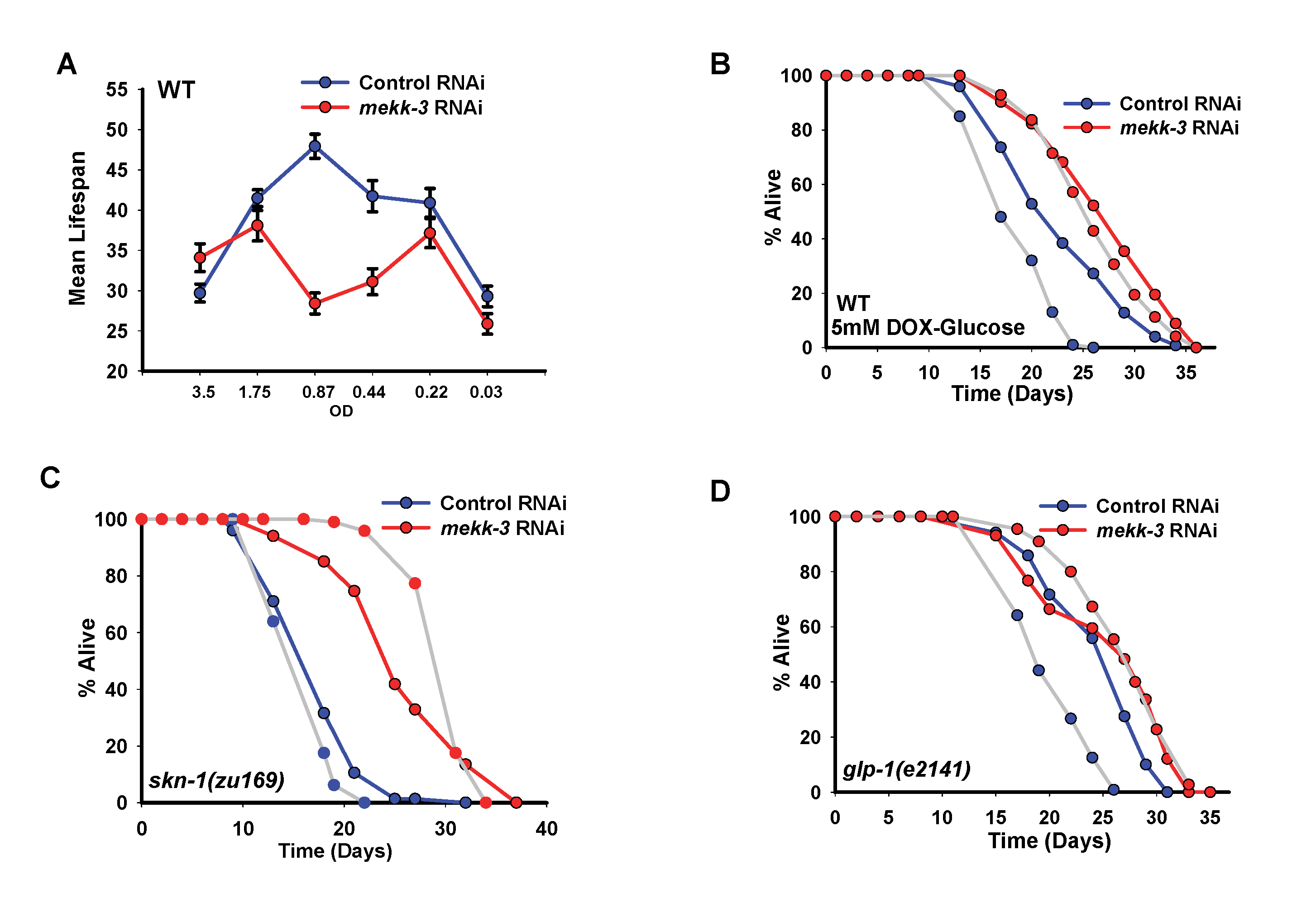

Supplement: Supplementary file 5 — Fig. S5 (A) WT worms grown on control or mekk-3 RNAi were transferred to liquid DR culture (see Data S1) having different concentrations of HT115 bacteria. The typical bell-shaped curve obtained after exposing WT worms, that were grown on control RNAi, to DR is absent in mekk-3 RNAi-treated worms. Mean lifespan is plotted against decreasing concentration of bacteria; error bars represent SEM. (B) The mekk-3 RNAi is unable to increase the lifespan of WT worms treated with 2-deoxy glucose (DOG) to the extent observed in untreated worms. In presence of 5 mm 2-deoxy glucose, MLS of WT on control RNAi was 23.91 ± 0.63 days (n = 135), on mekk-3 RNAi was 26.59 ± 0.65 days (n = 150) P < 0.0009. The grey lines indicate untreated WT worms grown on control or mekk-3 RNAi. (C) Lifespan of skn-1(zu169) on mekk-3 RNAi. MLS on control RNAi was 17.86 ± 0.49 days (n = 76), on mekk-3 RNAi was 26.39 ± 0.79 days (n = 67) P < 0.0001. The grey lines indicate WT grown on control or mekk-3 RNAi. Lifespans were performed at 20 °C. (D) The mekk-3 RNAi did not further increase the lifespan of glp-1(e2141). MLS on control RNAi was 24.83 ± 0.42 days (n = 120), on mekk-3 RNAi was 25.65 ± 0.55 days (n = 116) P = 0.0003. The grey lines indicate WT grown on control or mekk-3 RNAi. WT and glp-1(e2141) worms were hatched at 25 °C and transferred to 20 °C after 24 h. Lifespans were performed at 20 °C. [file acel0013-0641-sd5.tiff]

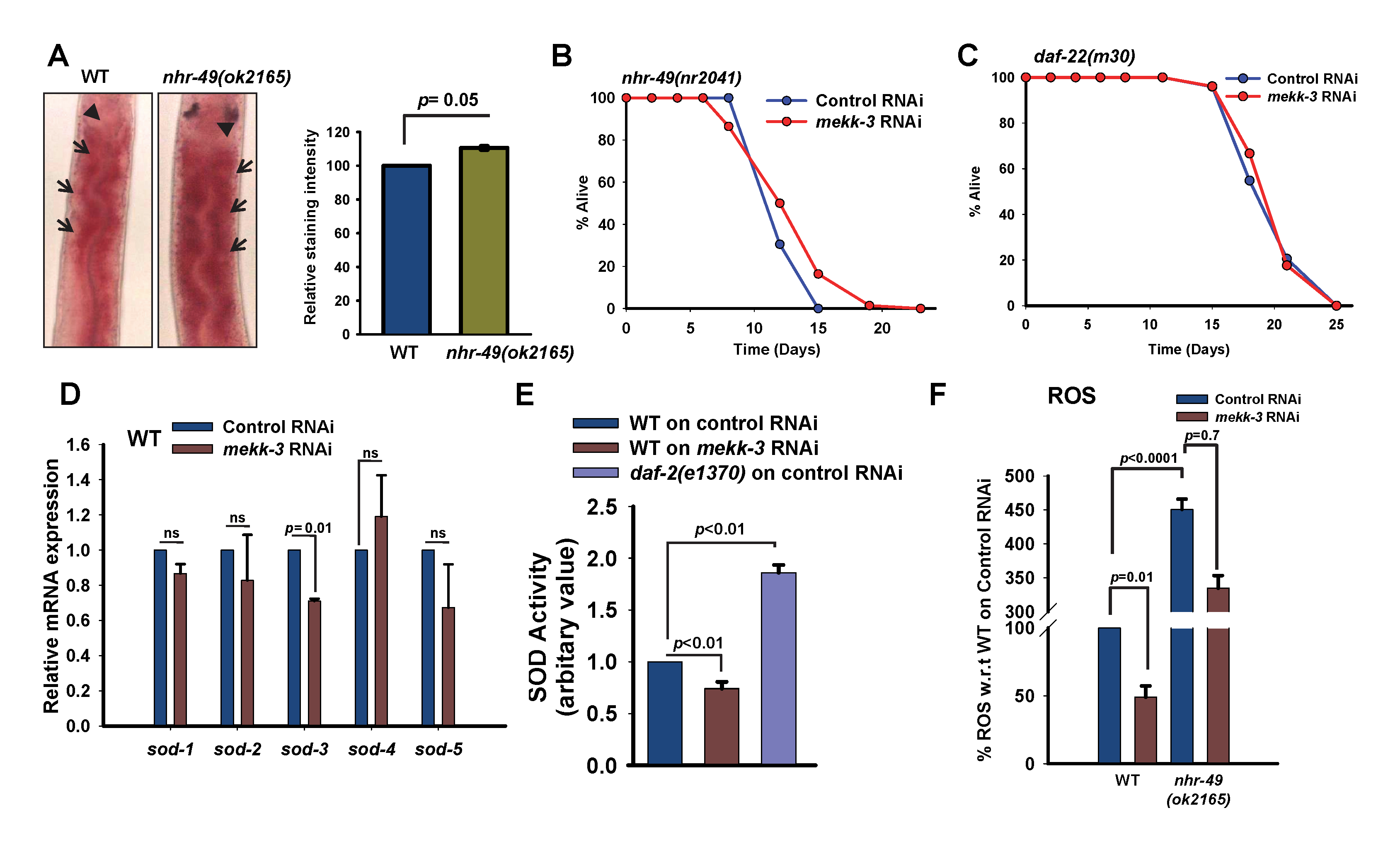

Supplement: Supplementary file 6 — Fig. S6 (A) nhr-49(ok2165) worms have higher amount of stored fat as indicated by Oil red O staining. Arrow head indicates the pharynx. Arrows highlight areas showing differences in hypodermal/intestinal fat staining between control and mekk-3 RNAi. Quantitation of staining is shown in the right panel. Error bar- SEM; n > 50; Student’s t-test. (B) mekk-3 RNAi was not able to increase lifespan in nhr-49(nr2041) to the same extent as in WT. MLS on control RNAi was 12.91 ± 0.12 (141), on mekk-3 RNAi was 13.67 ± 0.29 (140), P < 0.0006. Lifespans were performed at 20 °C. (C) mekk-3RNAi failed to increase lifespan in daf-22(m30). MLS on control RNAi was 20.34 ± 0.33 (n = 73), on mekk-3 RNAi was 20.59 ± 0.36 (n = 51), P = 0.6539. Lifespans were performed at 20 °C. (D) The expression of sod genes did not increase significantly between control- and mekk-3 RNAi-treated WT worms. The data for each of the sod genes is normalized to its expression in control RNAi-treated worms. Error bars indicate standard deviation over five independent biological replicates. P values- Student’s t-test between control and mekk-3 RNAi-treated worms for each gene. (E) The total SOD activity did not increase when WT worms were grown on mekk-3 RNAi. A daf-2(e1370) mutant had high levels of SOD activity. Graph represents average of 3 biological replicates; *P < 0.01, Student’s t-test between indicated samples. (F) mekk-3 RNAi decreased ROS in WT but failed to decrease it in nhr-49(ok2165). Average of three biological replicates. Error bars indicate standard deviation. Student’s t-test between samples indicated by lines. [file acel0013-0641-sd6.tiff]

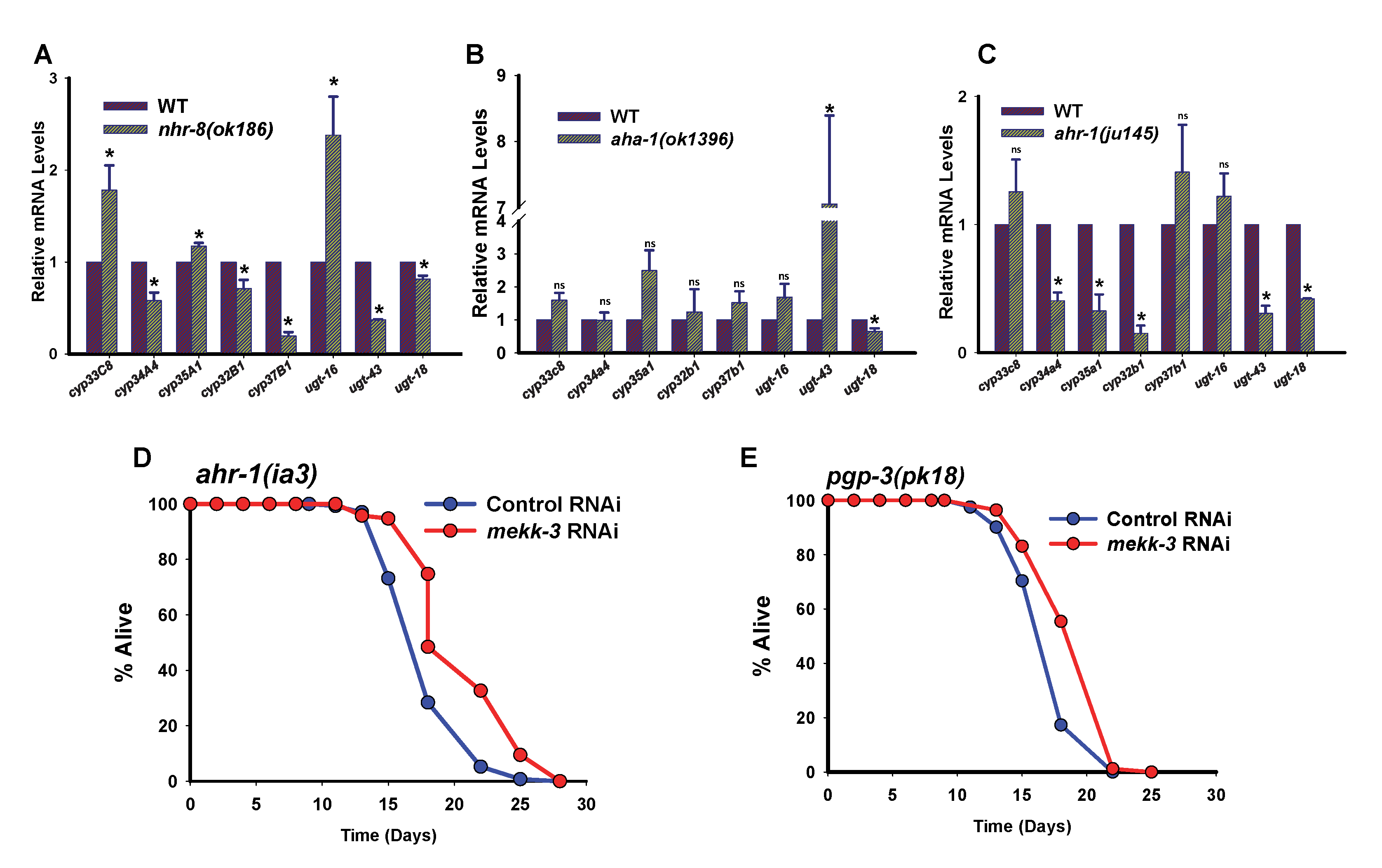

Supplement: Supplementary file 7 — Fig. S7 The expression of several xenobiotic detoxification genes are deregulated in (A) nhr-8(ok186), (B) aha-1(ok1396) and (C) ahr-1(ju145) mutants. Error bars- Standard deviation; P < 0.05, ns- not significant; Student’s t-test between WT and respective mutants for each gene. (D) Lifespan of ahr-1(ia3) on mekk-3 RNAi. MLS on control RNAi was 18.43 ± 0.27 days (n = 134), on mekk-3 RNAi was 20.96 ± 0.41 days (n = 95), P < 0.0001 by Log rank test. (E) Lifespan of pgp-3(pk18) on mekk-3 RNAi. MLS on control RNAi was 17.56 ± 0.30 days (n = 81), on mekk-3 RNAi was 19.67 ± 0.32 days (n = 83), P < 0.0001. All lifespans were performed at 20 °C. [file acel0013-0641-sd7.tiff]

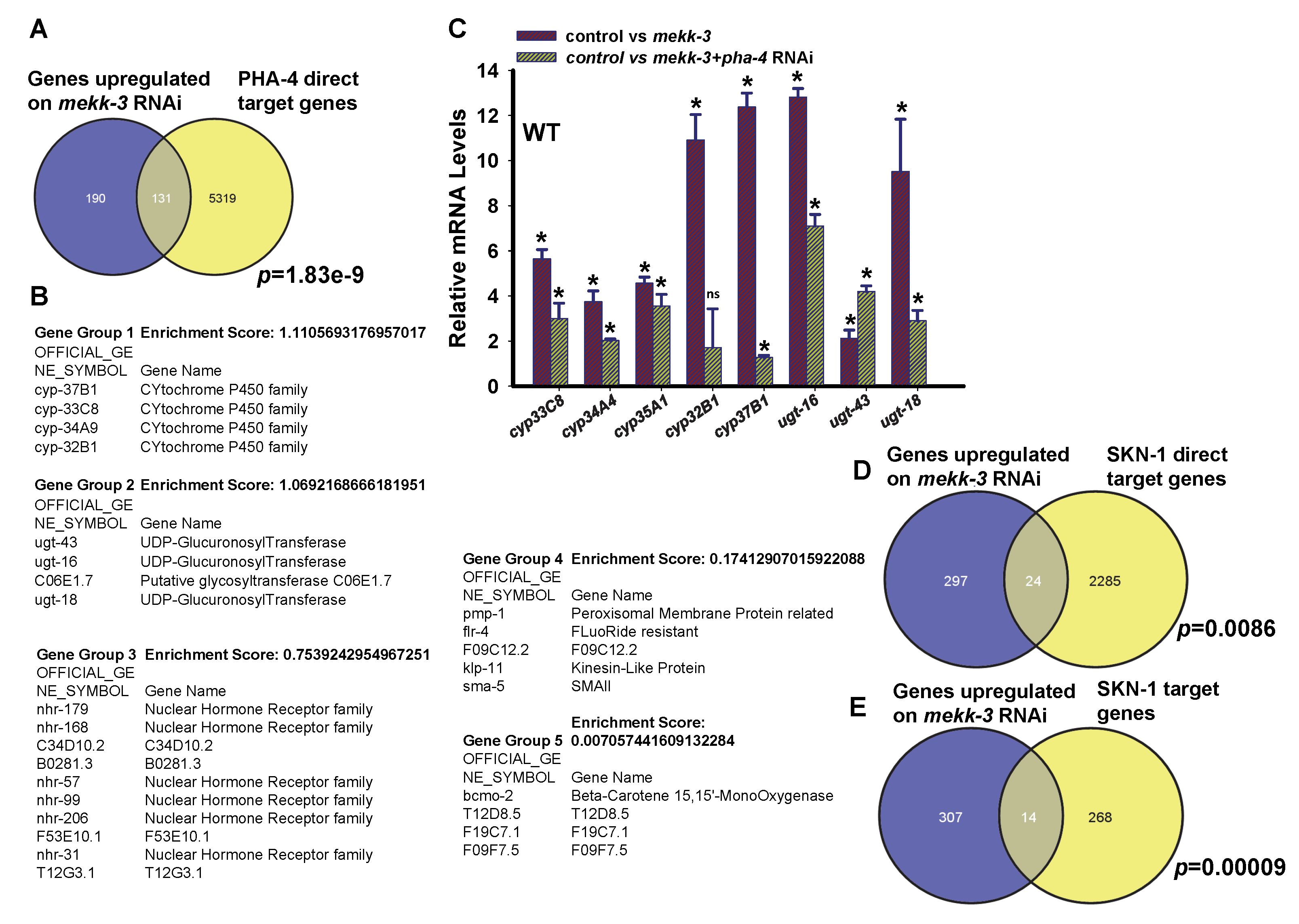

Supplement: Supplementary file 8 — Fig. S8 (A) Venn diagram showing the overlap of PHA-4 direct target genes (ModENCODE) that are upregulated when WT worms are grown on mekk-3 RNAi (overlap 40.8%, P = 1.83e-9, hypergeometric test). See Table S2 for gene-list. (B) Categorization of the PHA-4 target genes that are upregulated on mekk-3 RNAi using DAVID (david.abcc.ncifcrf.gov). (C) Several xenobiotic detoxification genes that are upregulated on mekk-3 RNAi in WT worms are downregulated when pha-4 is also knocked down. Worms were either grown on Control RNAi, mekk-3 RNAi or initiated on mekk-3 RNAi and transferred to pha-4 RNAi post-L4. Error bars- Standard deviation; P < 0.05, ns- not significant; Student’s t-test between WT grown on control RNAi vs. ones grown on mekk-3 or mekk-3 + pha-4 RNAi for each gene. (D) Venn diagram showing the overlap of direct target genes of SKN-1 (ModENCODE) that are upregulated when WT worms are grown on mekk-3 RNAi (overlap 7.5%, P = 0.0086, hypergeometric test). See Table S3A for gene-list. (E) Venn diagram showing the overlap of SKN-1 target genes (from microrarray, Oliveira et al., 2009) that are upregulated when WT worms are grown on mekk-3 RNAi (overlap 4.3%, P = 0.00009, hypergeometric test). See Table S3B for gene-list. [file acel0013-0641-sd8.tiff]

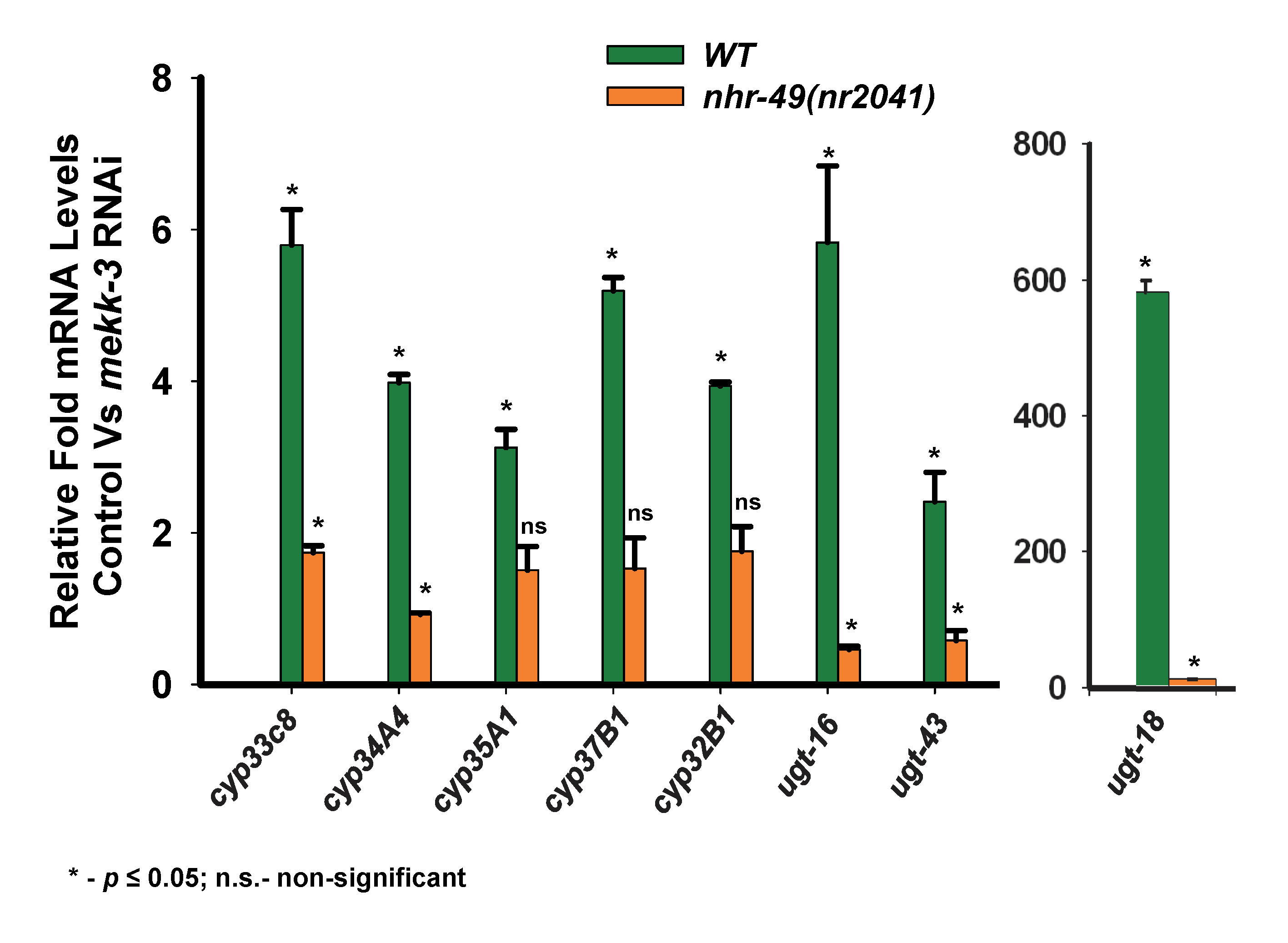

Supplement: Supplementary file 9 — Fig. S9 Transcript levels of select phase I and II xenobiotic detoxification genes were upregulated in WT worms undergoing mekk-3 knock-down-mediated DR (Green bar). [file acel0013-0641-sd9.tiff]

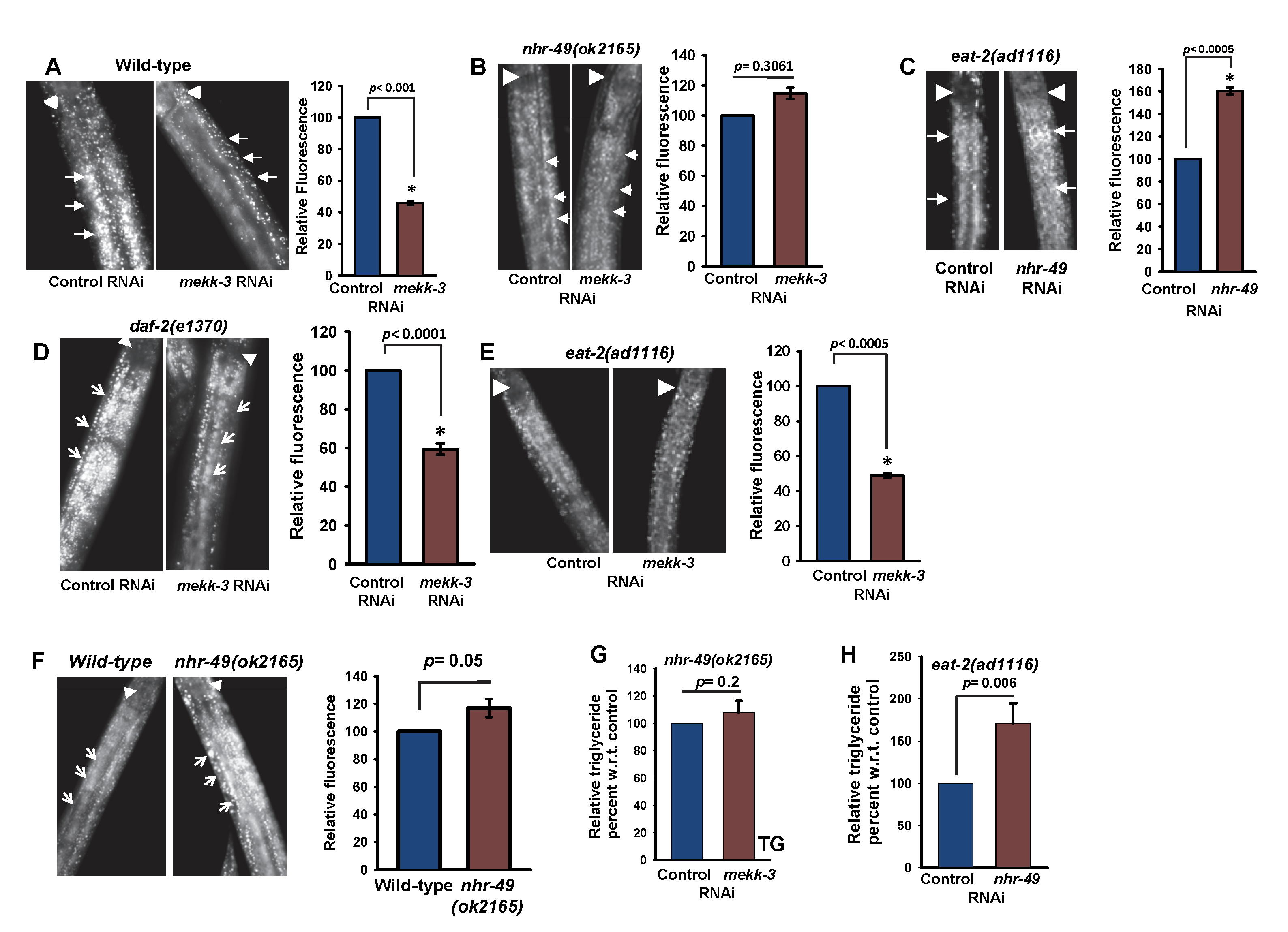

Supplement: Supplementary file 10 — Fig. S10 Nile Red staining of fixed WT or mutant worms grown on Control or specific RNAi. Left panel shows representative photo of worms while right panel shows quantification of fluorescence. [file acel0013-0641-sd10.tiff]

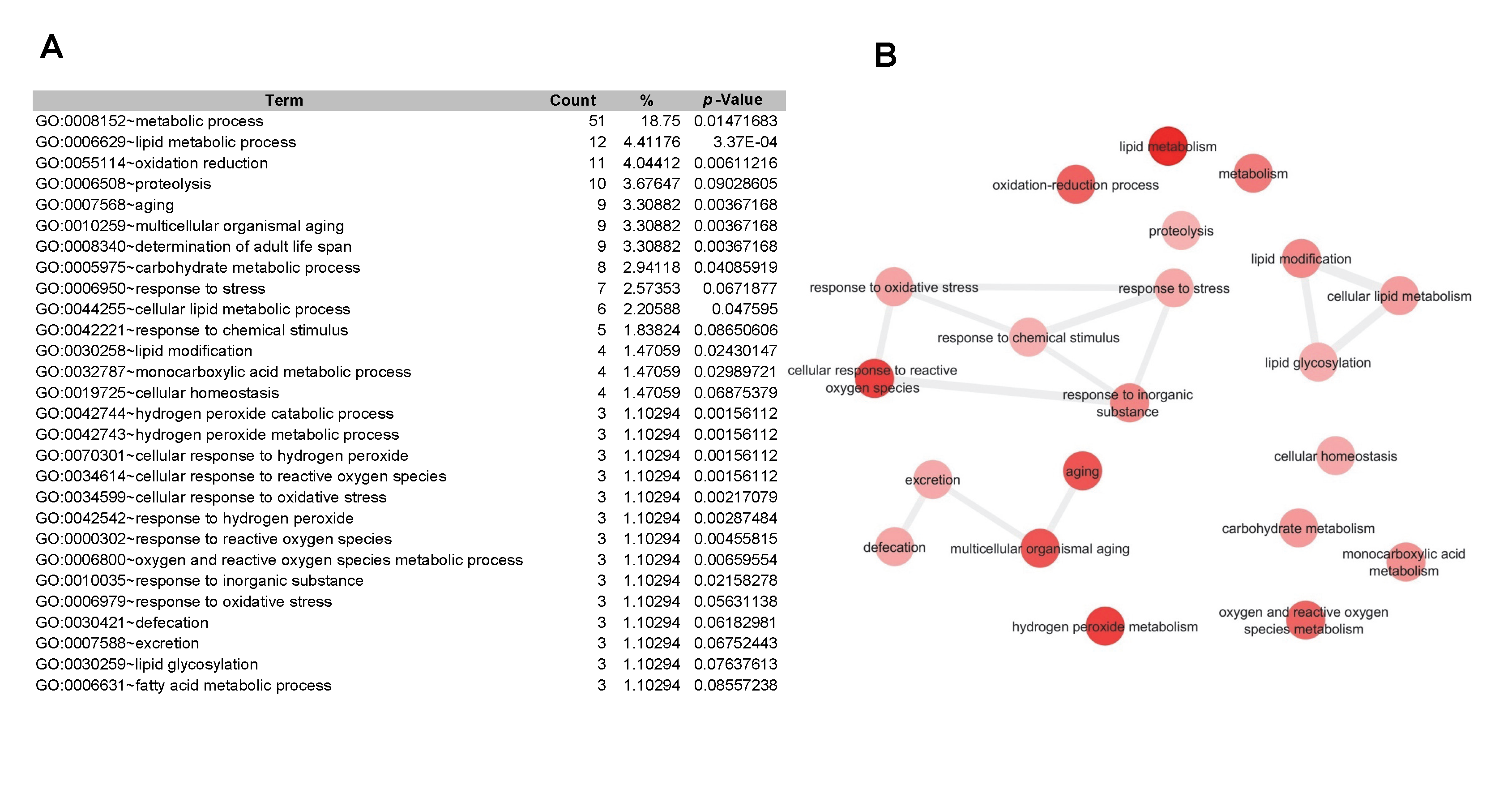

Supplement: Supplementary file 11 — Fig. S11 The genes upregulated when WT worms were grown on mekk-3 RNAi were categorized on the basis of GO term using DAVID and REVIGO softwares; details in Data S1. [file acel0013-0641-sd11.tiff]

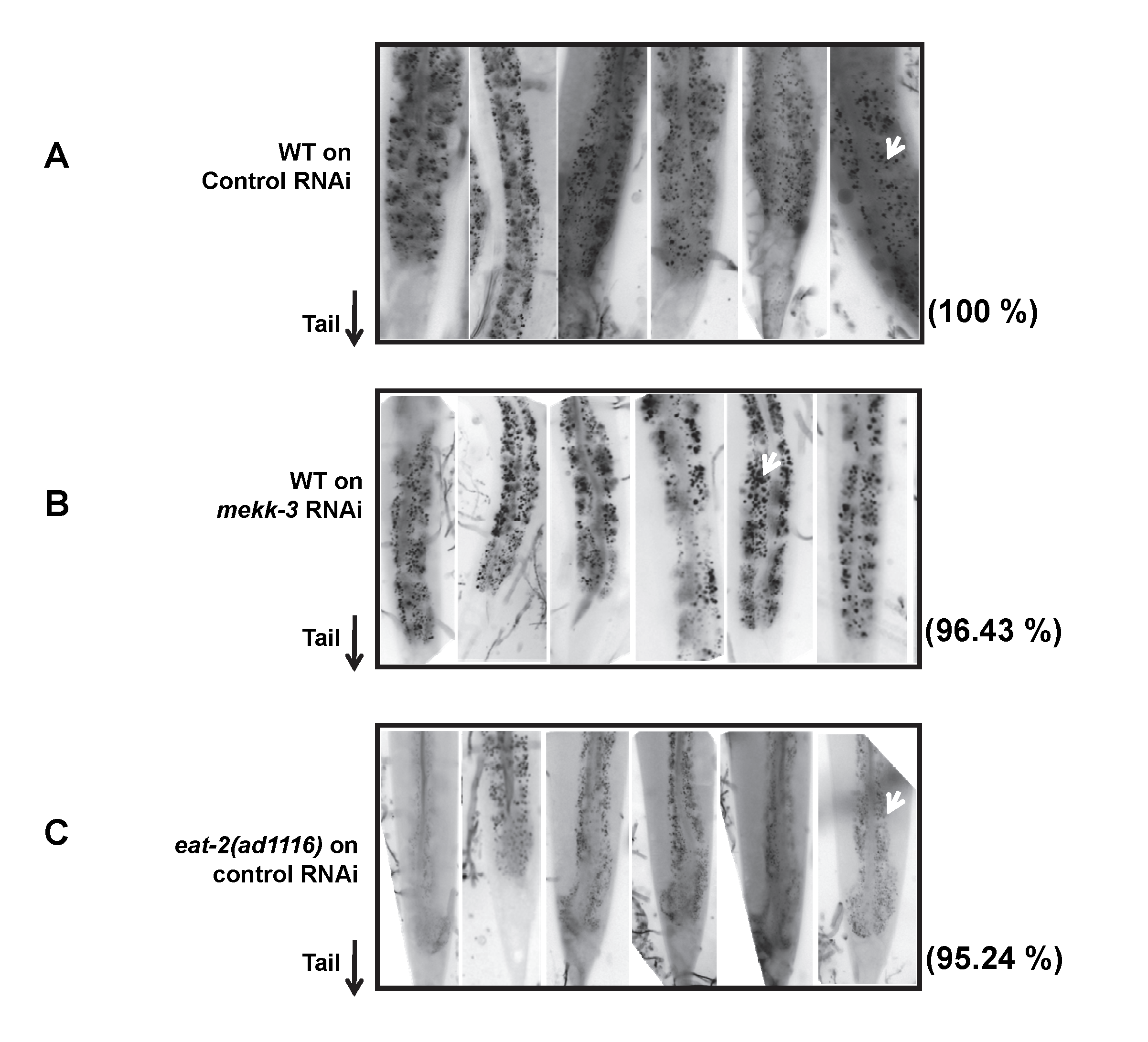

Supplement: Supplementary file 12 — Fig. S12 Wild-type worms on Control or mekk-3RNAi as well as eat-2(ad1116) grown on control RNAi were fed BODIPY containing bacterial feed for 20 min. [file acel0013-0641-sd12.tiff]

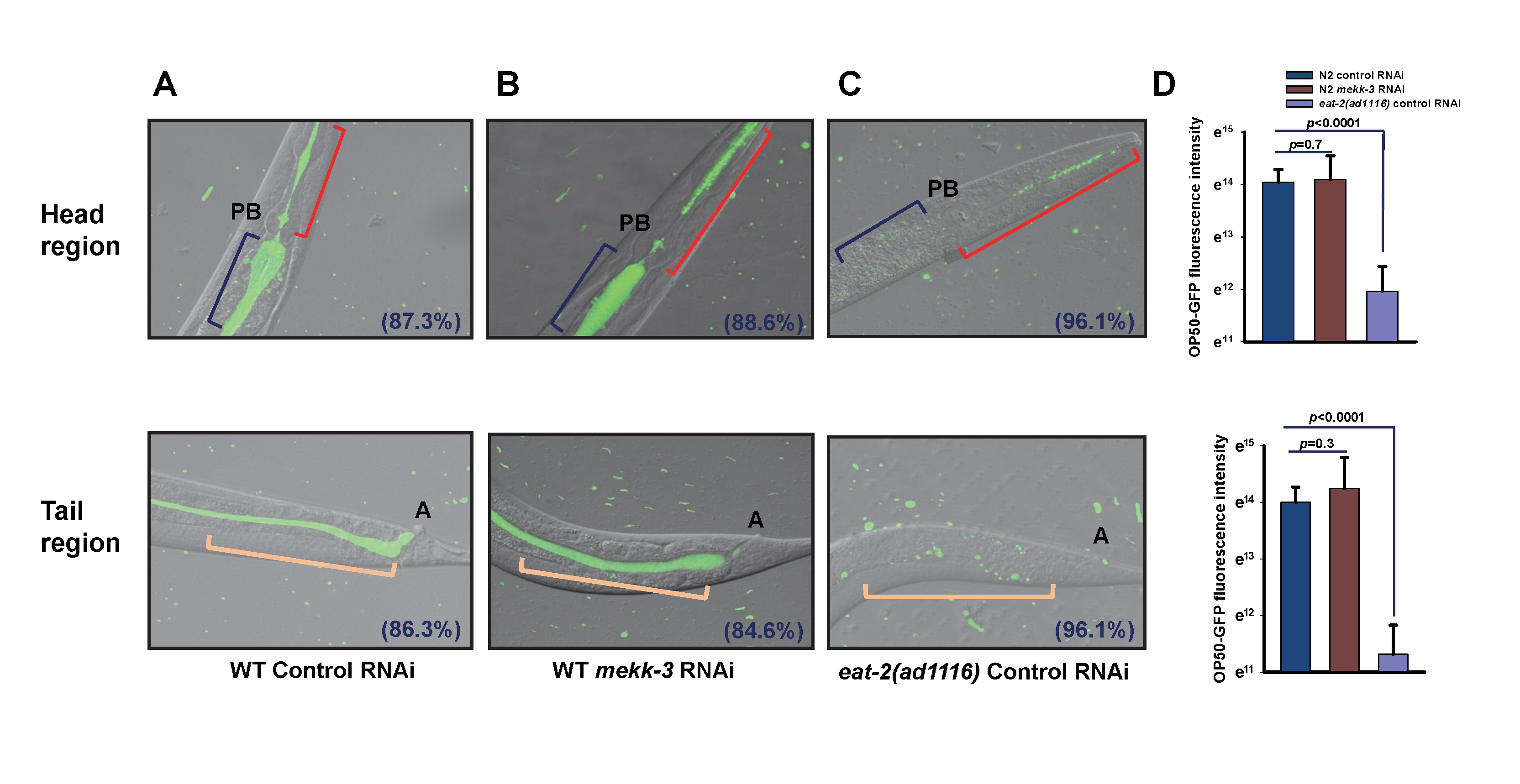

Supplement: Supplementary file 13 — Fig. S13 The mekk-3 RNAi worms have normal bacterial food intake. [file acel0013-0641-sd13.tiff]
