# Supplementary material for: A novel kinase regulates dietary restriction-mediated longevity in Caenorhabditis elegans
Source: Aging Cell. 2014 Mar 21;13(4):641–55. doi: 10.1111/acel.12218 (PMC4326946; doi:10.1111/acel.12218)
Supplement: Supplementary file 14 — Data S1 Experimental procedures. [file acel0013-0641-sd14.doc]

**Supplementary experimental procedures:**

***Strain maintenance***

Unless otherwise mentioned, all strains were maintained at 20 ^o^C using standard *C. elegans* techniques (Stiernagle 2006). Unless mentioned explicitly, all RNAi experiments were initiated from eggs. Strains used in the study are: N2 Bristol as wild-type, *daf-2(e1370)III, daf-2(e1368) III, daf-16(mgDf50) I, hsf-1(sy441) I, daf-3(mgDf90) X, daf-5(e1386)II, daf-7(e1372) III, daf-2(e1370) III;daf-3(mgDf90)X, eat-2(ad1116) II, eat-2(ad1113) II, eat-2(ad465) II, clk-1(qm30)III, skn-1(zu169) IV/nT1[unc-?(n754) let-?](IV;V), smg-1(cc546) I, smg-1(cc546) I; pha-4(zu225) V, nhr-49(ok2165) I, nhr-49(nr2041) I, mev-1(kn1)III, gas-1(fc21) X, nhr-8(ok186) IV, aha-1(ok1396) I/hT2[bli-4(e937) let-?(q782) qIs48](I;III), ahr-1(ju145) I, ahr-1(ia3) I, pgp-3(ok3091) X, pgp-3(pk18)X, daf-22(m130)II, rde-1(ne219) V, rde-1(ne219) V; kzIs9, rde-1(ne219) V; kzIs20, rde-1(ne213) V; kbIs7, glp-1(e2141) III, fer-15(b26) II; fem-1(hc17) IV*.

***Kinase assay***

The *mekk-3* cDNA was cloned into p3XFLAG-Myc-CMV-26 (Sigma, USA). The glutamate to alanine substitution (E123A) was brought about by site-directed mutagenesis (Stratagene, USA). COS-1cells at 40% confluency were transfected with 2 μg of each plasmid in 6-well tissue culture plates using LipofectAMINE (Life Technologies Inc., USA). Forty eight hours after transfection, cells were lysed in the lysis buffer (25 mM Tris-HCl, 300 mM NaCl, and 1% Triton X-100). The MEKK-3 protein was immunoprecipitated using anti-Flag M2 antibody (Sigma, USA) and washed 5 times in kinase buffer (10 mM HEPES-KOH, pH 7.5, 5 mM MgCl2, and 5 mM CaCl2). For *in vitro* kinase assays, the immunoprecipitated protein samples were divided into two aliquots. One aliquot was analyzed by western blotting using anti-Flag antibody. The second aliquot was used for kinase assay using MBP as a substrate at 30 ^o^C for 15 min in a kinase buffer containing 10 μCi of 32P-ATP (10 Ci/mmol) and 10μM ATP. The reaction was terminated by adding an equal volume of 2x SDS sample buffer (80 mM Tris, pH 6.8, 3.2% SDS, 16% glycerol, 200 mM dithiothreitol, 0.02% bromphenol blue) and subjected to SDS-PAGE followed by autoradiography.

***Preparation of RNAi plates***

RNAi plates were prepared by supplementing Nematode Growth Media (NGM) media with 100 µg/ml ampicillin and 2 mM IPTG. RNAi bacteria were grown overnight at 37°C in LB media supplemented with 100 µg/ml ampicillin and 12.5 µg/ml tetracycline. The next day, the cultures were diluted (1:50) in LB containing 100 µg/ml ampicillin and grown at 37°C until an OD_600_ of 0.6. The bacterial pellets were resuspended in 1X M9 buffer containing 1 mM IPTG and 100 µg/ml ampicillin. About 200 µl of the bacterial suspension was seeded onto the RNAi plates. The seeded plates were dried at RT for 2-3 days and stored at 4°C till further use.

***RNAi lifespan***

Strains for RNAi life span assays were initially grown on *E. coli* OP50. Gravid adult worms were bleached and the eggs were hatched on plates containing respective RNAi bacteria. L4 or young adult worms were transferred to an intermediate RNAi plate for 12 hours and then onto RNAi plates overlaid with 5-fluorodeoxyuridine (FUDR) to a final concentration of 0.1 mg/ml (Hoso*no et a*l. 1982). Worms were scored as dead or alive by tapping them with a platinum wire every 2-3 days. Worms that were sick or died from vulval bursting were censored. Statistical analyses for survival were conducted using Mantel-Cox log rank test using OASIS software available at http://sbi.postech.ac.kr/oasis (Ya*ng et a*l. 2011). Life span is expressed as average life span ± SEM. Full data and an additional biological repeat are reported in Table S1.

For temporal requirement experiments, eggs were hatched on Control RNAi plates and worms were transferred to *mekk-3* RNAi plates at L1, L2, L3, L4 or YA stages. FUDR was overlaid on the plates when worms reached L4.

For life spans with 2-Deoxy glucose (2DG), NGM RNAi plates were poured along with 5mM final concentration of 2DG. The plates were dried and seeded with RNAi bacteria as usual.

***Preparation of stock solution for Nile red and Oil Red O***

A Nile Red stock of 1 mg/ml was diluted to 100 ng/ml using water. Oil Red O stain was prepared as a 5mg/ml stock in isopropanol and equilibrated on a rocker shaker for several days. The working stock of Oil Red O was prepared by diluting the equilibrated stock to 60% using water and allowed to stand for 10 min following which it was filtered using a 0.22µm filter.

***Fat Staining***

Fat storage was determined in fixed worms using Oil Red O or Nile Red (O'Rour*ke et a*l. 2009; Y*en et a*l. 2010). Briefly, worms were synchronized by hypochlorite treatment and strains were grown on different RNAi plates till L4-YA stage. The worms were washed and resuspended in 120µl 1X PBS. To this an equal volume of 2X MRWB buffer (160mM KCl, 40mM NaCl, 14 mM Na_2_EGTA, PIPES pH 7.4, 1mM Spermidine, 0.4mM Spermine, 2% Paraformaldehyde, 0.2% beta-mercaptoethanol) was added and incubated with shaking for 45 minutes. The worms were subjected to three freeze-thaw cycles in dry ice/ethanol bath, pelleted and washed with 1XPBS. The working solution of stains were added to the fixed worms and incubated overnight on a shaker at room temperature. Following overnight staining, worms were washed twice with 1X PBS and mounted on 2% agarose slides for visualization using a AxioImager M2 microscope (Carl Zeiss, Germany) fitted with Axiocam MRm camera.

***Body bends Assay***

Worms were transferred to a bacteria-free plate on the day of scoring. Each worm was gently prodded on the tail with a platinum wire and total number of body bends was counted for a 20 second time span. The experiment was performed on day 2, day 6 and day 10 of adulthood. A total of around 20 worms were scored per RNAi on each day. A body bend was scored each time the region just posterior to the pharynx reached a maximum bend in the opposite direction from the one counted last. Mobility is expressed as body bend/20 sec ± Std. Dev.

***Pharyngeal pumping and food intake***

Pharyngeal behaviour and food intake were studied following modified protocols from previously described work (Raiz*en et a*l. 2005). Pharyngeal pumping was counted in young adult worms. A 10 sec video of each worm was taken using Axiocam MRm camera attached to M205FA microscope (Leica, Germany). The video was slowed down and pharyngeal pumping was counted for the 10 sec period. Around 10-15 worms per treatment were analysed. Assays were repeated at least three times.

To quantify food intake, WT L4 larvae grown on control or *mekk-3* RNAi were placed on NGM plates seeded with a 250:1 (vol:vol) mixture of HT115 bacteria and Fluoresbrites Multifluorescent microspheres (0.2 μm diameter, Polyscience Inc., USA). The worms were allowed to feed for 10 min and then washed twice with M9 buffer. The worm pellet was resuspended in 30 μl buffer and transfer to a freshly made 2% agarose pad. The worms were kept under an AxioImager M2 microscope and photographed. Fluorescent intensity of 10 worms was measured using NIH ImageJ software.

For visualizing fatty acid uptake, BODIPY-conjugated fatty acid (Invitrogen D3823) dissolved in DMSO (1mg/ml) was used at a final concentration of 2.47 mM. For preparation of BODIPY containing feed, one ml of overnight grown *E.coli* OP50 culture was pelleted down at 12000 rpm for 2 min. Supernatant was discarded and pellet was resuspended in 1 ml of S-basal buffer. To this, 1.5 μl of BODIPY was added and mixed. Synchronized L4 worms grown on respective RNAi were washed 2-3 times in S-basal to remove any adhering bacteria. Worms were than resuspended in 500 μl of the feed and incubated at RT for 20 min with continuous rotation at room temperature. At the end of 20 min, tubes were placed on ice and worms were allowed to settle at bottom of tube. About 5 μl of this was pipetted out and placed on a glass slide with agarose pad. A 20mM sodium azide solution was used to anaesthetize the worms. Fluorescence images were captured under microscope using similar exposure time and settings.

***Triglyceride quantification:***

For triglyceride quantification, 150 L4 hermaphrodite worms grown on control or *mekk-3* RNAi at 20 ^o^C were collected in 100ul of S-basal media. Worms were washed thrice with S-basal to remove any attached bacteria. An equal volume of a 5% Triton X-100 solution with 1x protease inhibitor EDTA-free (Sigma Aldrich, USA) was added to this. Worms were sonicated using Bioruptor (Diagenode, USA) at maximum intensity for 6 cycles (30 sec on and 30 sec off pulse). Lipids were dissolved by heating worm lysates at 90 ^o^C for 5 minutes with brief vortexing after every 1 minute. This step was repeated twice to ensure complete mixing of lipids. Lysate was centrifuged at 5000 rpm for 3 minutes and supernatant collected in separate tubes. This supernatant was then used for the triglyceride assay as per the manufacturer’s instructions (Biovision, USA).

***Autophagy***

Transgenic worms expressing *lgg-1::gfp* (Lapier*re et a*l. 2011) were grown for two generations at 20°C on control or *mekk-3* RNAi. The second generation L3 larval stage worms were anesthetised with 20 mM Sodium azide on 2% agarose slides and scored for GFP puncta in their hypodermal seam cells. Between 3-10 seam cells were scored for each of the 5-15 worms examined. The average puncta per seam cells of each worm was calculated. Standard deviation was calculated between average puncta per seam cells of all the worms scored. Assay was repeated three times.

***Muscle integrity measurements***

Age-related deterioration of *C. elegans* body wall muscle was analyzed using *ccIs4251* [pSAK2 (*myo-3::NGFP-LacZ*)], hereafter referred to as *myo-3::gfp* worms. These worms were grown on Control or *mekk-3* RNAi plates supplemented post-adulthood with FUDR. On day 13 of adulthood, the worms were immobilized on glass slides coated with 2% agarose in presence of 20 mM sodium azide. The worms were visualized using AxioImager M2 microscope (Carl Zeiss, Germany) fitted with Axiocam MRm. For each RNAi, several nuclei of 6-10 worms were photographed. Each muscle nucleus was scored as ‘intact’, ‘moderate’ or ‘severe’ depending on their morphology. A nucleus was scored as ‘intact’ if it had intact membrane with no signs of degradation, ‘moderate’ when the nuclear membrane had begun to disintegrate but the nucleoplasm showed no or very little dark patches while ‘Severe’ represented all those nuclei which had increased nucleolar size, dark patches in the nucleoplasm, distorted appearance and membrane disintegration.

***Lipofuscin autofluorescence***

Lipofuscin autofluorescence in the gut was evaluated on day 2, 5, 10 and 15 of adulthood. WT worms were grown on Control RNAi or *mekk-3* RNAi until L4. Around 20-30 worms were then transferred (in triplicate) to fresh RNAi plates supplemented with FUDR. To determine lipofuscin autofluorescence, adult hermaphrodites were anesthetized in 0.2% Sodium Azide and mounted on 2% agarose pads for visualization under microscope using FITC filter. Images were captured using a constant exposure time as above.

***Brood size and reproductive span***

Wild-type worms were grown on control or *mekk-3* RNAi till late L4. Five worms were picked onto fresh RNAi plates and allowed to lay eggs for 24 hours. The worms were then transferred to fresh plates everyday and the eggs/L1s on previous day’s plate were counted. Only eggs that produced viable progeny were considered. Data is presented as number of progenies ± Std. Dev. For calculating reproductive span, individual worms were transferred to fresh plates every day and scored for viable progenies. Fraction of worms that laid viable progenies were plotted against number of days.

***RNA isolation***

RNA was isolated using Trizol (Invitrogen, USA). Briefly, worms grown on vector or RNAi of interest were washed off the plates with M9 buffer. Then, 0.3 ml of Trizol reagent was added and the worms lysed by vigorous vortexing. RNA was purified by phenol:chloroform:isoamylalcohol extraction and ethanol precipitation. The concentration and the purity of the RNA were determined using Bioanalyzer (Agilent, USA). Alternatively, the quality of the ribosomal 28 S and 18 S on an agarose gel was used as a measure of integrity and the absorbance at 260/280 nm was used to determine quantity.

***Cloning***

*mekk-3 cDNA* (1.34 kb) was amplified from wild-type cDNA pool using primers listed below. *PstI* and *XbaI* sites were incorporated in the 5’ and the 3’ primers, respectively. The amplified product was cleaned using PCR Purification Kit (Qiagen, USA) and digested using *PstI* and *XbaI*. This fragment was then cloned in the same sites of *pL4440* vector.

***Generation of transgenic animals***

Transgenic worms were generated using a Microinjection setup consisting of Nikon TiS inverted microscope fitted with Eppendorf Femtojet Express and Transferman NK2. The 2.9 kb promoter region upstream of start codon of *mekk-3* was amplified using a forward primer (5’CCCAAGCTTTGCATTCAGAGGAGAAATACT3’) and reverse primer (5’GCTCTAGATGTTGCTGTATAAACATATTTTGACGT3’) carrying *HindIII* and *XbaI* restriction sites, respectively, using Expand Long Template system (Roche, Germany) and cloned into pPD95.75. The recombinant plasmid was linearized using *HindIII*, purified using PCR purification kit (Qiagen, USA) and injected at a concentration of 25 ng/μl into the syncytial gonad of wild-type worms along with 50 ng/μl pRF4 *rol-6* co-injection marker. Transformants were selected based on rolling phenotype, and GFP expression was observed under a fluorescence microscope. Four independent lines carrying extra chromosomal arrays were obtained and all gave similar patterns of GFP expression.

***Microarray and qRT-PCR analysis***

cDNA was synthesized using 2.5 µg of RNA and the SuperScript III cDNA synthesis kit (Invitrogen, USA) according to manufacturer’s specifications. Microarray analysis was carried out commercially at Genotypic Technology (Bangalore, India) using an Agilent, USA platform. Data was analysed using GeneSpring software (Agilent, USA) or CLC Genomics Workbench 4 (CLC Bio, USA) and more than 2 fold expression changes with *p* value <0.05 is reported. The microarray data is available at GEO repository with Series record number GSE40252. GO analysis was performed using DAVID (Denn*is et a*l. 2003) and REVIGO (Sup*ek et a*l. 2011).

Gene expression levels were determined by quantitative real time PCR (qRT-PCR) using the Mesagreen MasterMix (Eurogentec, Belgium) and Realplex PCR system (Eppendorf, USA) according to manufacturer’s specifications. Relative gene expression, normalized to actin, was determined between worms treated with control RNAi and gene-of-interest RNAi. Statistical analysis was performed using SigmaPlot 10.0 (Systat software). All the primers used are listed in below.

***Oxidative stress***

Synchronized egg lay was performed using gravid adult worms that were grown on control or *mekk-3* RNAi. The progeny were allowed to develop at 20 °C, and FUDR was overlaid on the plates (final concentration of 50μg/ml) when worms turned young adults. At day 4-5 of adulthood, worms were collected from the RNAi plates and washed three times with S-complete buffer. Approximately 15 adults were dispensed into each well of a 24-well tissue culture plate containing 1ml of 0, 4, 20, 50, 100 or 200 mM paraquot (Sigma) in S-complete buffer. Data from 100 mM paraquot is reported here. Worms in the paraquot were scored every 2–3 h for survival. Worms that failed to respond to gentle prodding were scored as dead. Experiments were repeated two independent times and data is presented as mean survival ± SEM.

***ROS generation***

To measure intracellular levels of ROS, young adult worms were collected in M9 buffer and washed four times using the same buffer. Worm extracts were prepared by freezing the pellet in liquid nitrogen, freeze-thawing for three cycles and then sonicating (setting of 30 amplitude, 12 cycles of 1 sec pulse on/off, 5-8 times using Misonix Ultrasonic processor 4000) in 1× PBS. The cell extract was centrifuged at 20,000 *g* for 15 min at 4 °C, and the protein concentration of the supernatant was determined using Bradford protein estimation kit (Biorad, USA). Supernatant containing 5 μg of protein was pre-incubated with 50 μ M of 2,7-dichlorofluorescein diacetate (DCF-DA, Molecular Probes, USA) in 100 μL of PBS at 37 °C for 1 h. Fluorescence intensity was measured in FLUOstar Omega (BMG Labtech, USA) every 10 min for 1 h at excitation wavelength 485 nm and emission wavelength 520 nm. Data is presented as percent WT on control RNAi ± Std.Dev.

***SOD activity***

The total SOD activity was measured using commercially available kit (Sigma, USA). Worms grown on control or *mekk-3* RNAi were harvested and lysed using a waterbath sonicator (Diagenode, USA). SOD activity was measured according to manufacturer’s specifications. The data was normalized to total protein content.

**Identification of PHA-4 and SKN-1 targets in *mekk-3*microarray data**

ChIP-Sequencing peak data for all developmental stages of PHA-4:OP37 strain, having project IDs 582, 585, 3158, 3215, 2945, 4033, 2598 and 584, deposited by the Snyder lab, were downloaded from modENCODE (www.modencode.org)*.* Statistically significant peaks from these different datasets were clustered into one peak file and PeakAnalyzer (Salmon-Div*on et a*l. 2010) software was used to find genes having enriched PHA-4 binding sites in 2kb promoter region using annotations provided in C. elegans WBCEL235 release. The resulting target list was then compared to genes that were upregulated in mekk-3 RNAi treated worms. Similar approach was taken for SKN-1.

***Bacterial dilution-induced DR (BDR)***

***BDR media preparation***

HT115 bacteria were streaked on LB agar plates supplemented with ampicillin and tetracycline and incubated for 12 hour at 37°C. A single colony was inoculated in 200 ml of LB supplemented with ampicillin, in a 2 L flask  and grown for 12 hours at 37°C. The bacterial cells was collected by centrifugation at ~5000 rpm for 10 min. Cells were then re-suspended in S-basal/cholesterol/antibiotics solution [1× S-Basal Medium (5.85 g NaCl, 1.0 g K_2_HPO_4_, 6.0 g KH_2_PO_4_, 1.0 ml cholesterol (5 mg/ml in ethanol), Carbenicillin (50 µg/ml), Kanamycin (10 µg/ml), Tetracycline (1 µg/ml), Milli-Q water to one liter -sterile filtered] and diluted to the required optical density (OD) using S-basal. OD was measured at 600 nm. Diluted bacterial solutions were kept at 4°C for maximum 2 weeks. Range of O.D. used: 3.5, 1.75, 0.87, 0.44, 0.22, and 0.03.

***BDR Lifespan assays***

All experiments were carried out at 20°C. OP50-seeded NGM plates having well-fed gravid adult worms were bleached and eggs were then kept on a 60 mm NGM RNAi plates seeded with control or *mekk-3* RNAi. After the worms reached young adult stage, FUDR (100 mg/L) was added to each plate to arrest progeny development. About 24 hours following the addition of FUDR, worms were transferred to a single well of a 12-well cell-culture plate containing 1 ml (30 worms per well) of RT S-basal/cholesterol/antibiotics solution with FUDR (100 mg/L). The plate was kept on an Eppendorf Mix-mate 96 well shaker on a rotation of 300rpm, making sure that liquid did not spill. This was placed in a 20 °C incubator for one hour to remove any residual bacterial clumps adhered to the worms. During this time, bacterial solutions for the lifespan were added to the 12-well cell culture plates (1 ml solution per well along with FUDR at 100 mg/L). After one hour, 10 worms per well were moved from the S-Basal to the appropriate bacterial solutions using a P200 pipette. To prevent loss due to worms sticking to the pipette, glass pipette tips were used. These were made using Pasteur pipettes and were connected to the P200 using a short piece of rubber tubing. Worms were moved to fresh bacterial solutions after every 3-4 days at which point they were scored for movement using a platinum wire. Any worm that did not move was pipetted onto a Nematode Growth Media (NGM) plate and allowed to recover. Those that did not respond to gentle prodding with a worm pick were the scored as dead. Any responsive worm was returned to the experiment. During experiments, plates were maintained at 20°C in an Eppendorf 96 well shaker at rotation of 300 rpm.

**References:**

Dennis G, Jr., Sherman BT, Hosack DA, Yang J, Gao W, Lane HC, Lempicki RA (2003). DAVID: Database for Annotation, Visualization, and Integrated Discovery. *Genome Biol*. **4**, P3.

Hosono R, Mitsui Y, Sato Y, Aizawa S, Miwa J (1982). Life span of the wild and mutant nematode *Caenorhabditis elegans*. Effects of sex, sterilization, and temperature. *Exp Gerontol*. **17**, 163-172.

Lapierre LR, Gelino S, Melendez A, Hansen M (2011). Autophagy and lipid metabolism coordinately modulate life span in germline-less *C. elegans*. *Curr Biol*. **21**, 1507-1514.

O'Rourke EJ, Soukas AA, Carr CE, Ruvkun G (2009). *C. elegans* major fats are stored in vesicles distinct from lysosome-related organelles. *Cell Metab*. **10**, 430-435.

Raizen D, Song BM, Trojanowski N, You YJ (2005). Methods for measuring pharyngeal behaviors. *WormBook*, 1-13.

Salmon-Divon M, Dvinge H, Tammoja K, Bertone P (2010). PeakAnalyzer: genome-wide annotation of chromatin binding and modification loci. *BMC Bioinformatics*. **11**, 415.

Stiernagle T (2006). Maintenance of *C. elegans*. *WormBook*, 1-11.

Supek F, Bosnjak M, Skunca N, Smuc T (2011). REVIGO summarizes and visualizes long lists of gene ontology terms. *PLoS One*. **6**, e21800.

Yang JS, Nam HJ, Seo M, Han SK, Choi Y, Nam HG, Lee SJ, Kim S (2011). OASIS: online application for the survival analysis of lifespan assays performed in aging research. *PLoS One*. **6**, e23525.

Yen K, Le TT, Bansal A, Narasimhan SD, Cheng JX, Tissenbaum HA (2010). A comparative study of fat storage quantitation in nematode *Caenorhabditis elegans* using label and label-free methods. *PLoS One*. **5**, e12810.

List of primers used

| **Real Time Primers** | | |
| --- | --- | --- |
| **Gene name (Target)** | **Primer Name** | **Primer Sequence** |
| **Phase-I Detoxification** |  |  |
| ***cyp-34A4*** | **Forward Primer** | **GATTTGAACAGGGTGACCCAGAAT** |
|  | **Reverse Primer** | **TCGATGACATGCTCACCACT** |
| ***cyp-33C8*** | **Forward Primer** | **CGCTGGATGATGTGCTCAACTACTGG** |
|  | **Reverse Primer** | **GCTTCTTCTGCTCTTTCAGGTAGG** |
| ***cyp-32B1*** | **Forward Primer** | **GGTGTGTTGAAGTTATGGTTGGGACC** |
|  | **Reverse Primer** | **TGTCGCCGGTGCTGATTAAAAGAC** |
| ***cyp-35A1*** | **Forward Primer** | **GGAGGAATTGGATGCAAGATGTGCTG** |
|  | **Reverse Primer** | **TGGGGAGAAGACTTCAAACG** |
| ***cyp-37B1*** | **Forward Primer** | **GCTTGGAACGGGACTATTGAC** |
|  | **Reverse Primer** | **TTGTTCGAGGAAAACCTTGGCCTG** |
| **Phase-II Detoxification** |  |  |
| ***ugt-18*** | **Forward Primer** | **AACCGGC ACTGATAATT CCCCTTATGG** |
|  | **Reverse Primer** | **TAGAGCCCCATGTTCAACTGC** |
| ***ugt-16*** | **Forward Primer** | **CTTGCTGACGATCGACTAACC** |
|  | **Reverse Primer** | **CGGTCTGTATGGCTTCTCTAAG** |
| ***ugt-43*** | **Forward Primer** | **CTGGATATTCATCCG GGAGTTATGCG** |
|  | **Reverse Primer** | **TCAGTCTATCCAAAAGTCCCGGCATG** |
| ***Sod* Primers** |  |  |
| ***sod-1*** | **Forward Primer** | **AGGTCTCCAACGCGATTTTT** |
|  | **Reverse Primer** | **CCTGGTCATTTTCGGACTTC** |
| ***sod-2*** | **Forward Primer** | **CAACCGATCACAGGAGTCG** |
|  | **Reverse Primer** | **TTACAGGCTCCAAATCAGCA** |
| ***sod-3*** | **Forward Primer** | **GGAGTTCTCGCCGTCCG** |
|  | **Reverse Primer** | **GTCGAATGGGAGATCTGGGAG** |
| ***sod-4*** | **Forward Primer** | **ACGCGGTACTTCAGACCAAT** |
|  | **Reverse Primer** | **GAAGGGATGCTGTCGTTGTT** |
| ***sod-5*** | **Forward Primer** | **CCACAGGACGTTGTTTCCAA** |
|  | **Reverse Primer** | **ACCTTCGGCTTTCTGGGTAA** |
| ***F18F11.5*** | **Forward Primer** | **GGAAGGAAATACGCCAGATG** |
|  | **Reverse Primer** | **CCTGATGGTACATGGGCTCT** |
| ***actin*** | **Forward Primer** | **CTCTTGCCCCATCAACCATG** |
|  | **Reverse Primer** | **CTTGCTTGGAGATCCACATC** |
|  |  |  |
| **Cloning Primers** |  |  |
| **Gene name (Target)** | **Primer Name** | **Sequence** |
| ***F18F11.5* cDNA** | **Forward Primer** | **AACTGCAGGGCTAGTTATACAGTTACGGA** |
|  | **Reverse Primer** | **GCTCTAGATATTGCCCATTGTTGACTGGA** |
| ***F18F11.5* promoter** | **Forward Primer** | **CCCAAGCTTTGCATTCAGAGGAGAAATACT** |
|  | **Reverse Primer** | **GCTCTAGATGTTGCTGTATAAACATATTTTGACGT** |
| **Kinase assay primers** |  |  |
| **Gene name (Target)** | **Primer Name** | **Sequence** |
| ***F18F11.5* cDNA** | **Forward Primer** | **ATAAGAATGCGGCCGCGGCT AGT TAT ACA GTT ACG GAA TGG** |
|  | **Reverse Primer** | **TGCTCTAGATGC AAT CTT ATC AAC ACC ATA ACC** |
| ***F18F11.5* (E123A)** | **Forward Primer** | **ATT CTA CTG GCG GAT CCT TTT ATT GCA GGT CTC CCA AGT GCT** |
|  | **Reverse Primer** | **AGC ACT TGG GAG ACC TGC AAT AAA AGG ATC CGC CAG TAG AAT** |
|  |  |  |
| **cDNA subcloning primers** |  |  |
| ***F18F11*.*5* cDNA construct 1** | **Forward Primer** | **AACTGCAGGGCTAGTTATACAGTTACGGA** |
|  | **Reverse Primer** | **GCTCTAGACAGAAACCAGCTCTCTATGC** |
| ***F18F11.5* cDNA construct 2** | **Forward Primer** | **AACTGCAGGGCTAGTTATACAGTTACGGA** |
|  | **Reverse Primer** | **GCTCTAGATCCTCCAAAGCCGATAGATG** |
| ***F18F11.5* cDNA construct 3** | **Forward Primer** | **AACTGCAGGCATAGAGAGCTGGTTTCTG** |
|  | **Reverse Primer** | **GCTCTAGATATTGCCCATTGTTGACTGGA** |
